# MiR-494-3p regulates mitochondrial biogenesis and thermogenesis through PGC1-α signalling in beige adipocytes

**DOI:** 10.1038/s41598-018-33438-3

**Published:** 2018-10-10

**Authors:** Mengistu Lemecha, Katsutaro Morino, Takeshi Imamura, Hirotaka Iwasaki, Natsuko Ohashi, Shogo Ida, Daisuke Sato, Osamu Sekine, Satoshi Ugi, Hiroshi Maegawa

**Affiliations:** 1 Division of Endocrinology and Metabolism, Department of Medicine, Shiga University of Medical Science, Otsu, 520-2192 Japan; 2 Division of Molecular Pharmacology, Faculty of Medicine, Tottori University, Yonago, Japan; 3 Division of Pharmacology, Shiga University of Medical Science, Otsu, Japan

## Abstract

Mitochondria are critical in heat generation in brown and beige adipocytes. Mitochondrial number and function are regulated in response to external stimuli, such as cold exposure and β3 adrenergic receptor agonist. However, the molecular mechanisms regulating mitochondrial biogenesis during browning, especially by microRNAs, remain unknown. We investigated the role of miR-494-3p in mitochondrial biogenesis during adipogenesis and browning. Intermittent mild cold exposure of mice induced PPARγ coactivator1-α (PGC1-α) and mitochondrial TFAM, PDH, and ANT1/2 expression along with uncoupling protein-1 (Ucp1) in inguinal white adipose tissue (iWAT). miR-494-3p levels were significantly downregulated in iWAT upon cold exposure (*p* < 0.05). miR-494-3p overexpression substantially reduced PGC1-α expression and its downstream targets TFAM, PDH and MTCO1 in 3T3-L1 white and beige adipocytes (*p* < 0.05). miR-494-3p inhibition in 3T3-L1 white adipocytes resulted in increased PDH (*p* < 0.05). PGC1-α, TFAM and Ucp1 mRNA levels were robustly downregulated by miR-494-3p overexpression in 3T3-L1 beige adipocytes, along with strongly decreased oxygen consumption rate. PGC1-α and Ucp1 proteins were downregulated by miR-494-3p in primary beige cells (*p* < 0.05). Luciferase assays confirmed PGC1-α as a direct gene target of miR-494-3p. Our findings demonstrate that decreased miR-494-3p expression during browning regulates mitochondrial biogenesis and thermogenesis through PGC1-α.

## Introduction

Recent studies have elucidated the various critical processes underlying adaptation to cold environment through examining the transition of white adipocytes into beige/brite adipocytes^1^. White adipose tissue (WAT) is distributed throughout the body, and subcutaneous WAT accounts for about 85% of all body fat as the main storage organ of energy in a wide range of adiposity^2^.

Upon physiological stimuli such as chronic cold exposure or pharmacological treatment such as peroxisome proliferator-activated receptor γ (PPARγ) agonist β3-adrenergic receptor (β3-AR) stimulation, white adipocytes are re-programmed to beige adipocytes, which contain abundant mitochondria similar to brown adipose tissue (BAT), a major contributor of non-shivering thermogenesis^3,4^. PPARγ coactivator1-α (PGC1-α) directly links these external physiological stimuli to an internal metabolic response, such as mitochondrial biogenesis and function, via transcription factor A, mitochondria (TFAM), which regulates transcription and replication of mitochondrial DNA (mtDNA)^5,6^. Cold temperatures stimulate the sympathetic nervous system, leading to enhanced protein kinase A (PKA) signalling through β3-AR and cyclic AMP (cAMP), the basic pathway that drives thermogenesis^7^. Previous studies showed that uncoupling protein-1 (Ucp1) plays a significant role in the adaptive thermogenesis against cold through generation of heat by dissipating the proton-motive force over the mitochondrial membrane in BAT^8^. The reprogrammed beige adipocytes also respond to cAMP stimulation with high Ucp1 expression and respiration rates, as well as increased multilocular lipid droplets and increase in mitochondrial mass^9^. Mitochondrial biogenesis during beige differentiation has an impact on metabolism during the adaptive thermogenesis by beige cells^10^. The regulatory mechanisms underlying this process still remain unknown. However, some studies have demonstrated a link between non-coding microRNAs (miRNAs) in brown and beige fat development^11^.

MiRNAs are evolutionarily conserved, single stranded, non-protein-coding RNAs approximately 21–25 nucleotides in length that act as post-transcriptional gene regulators. MiRNAs inhibit target protein-coding genes through repressing messenger RNA (mRNA) translation^12^ by complementary binding to the 3′ untranslated region (3′-UTR) of their target mRNAs, which leads to mRNA degradation^13^. In mitochondria, miRNAs can provide a sensitive and rapid mechanism by which to regulate the expression of the mitochondrial genome in relation to the conditions and metabolic demands of the cell^14^. We previously reported that the expression of miR-494-3p was decreased during skeletal muscle differentiation and related with mitochondrial biogenesis in muscle differentiation and adaptation to exercise in skeletal muscle^15^. Because adipocytes and skeletal muscle cells share the same origin, mesenchymal stem cells, we hypothesized that miR-494-3p may play a role in adaptive thermogenesis in beige cells through mitochondrial biogenesis^16^. Therefore, here we examined this hypothesis using several beige adipocyte models.

## Results

### Cold exposure decreased miR-494-3p expression and increased major mitochondrial-related proteins during acute and chronic cold exposure in mice

**Figure 1:**
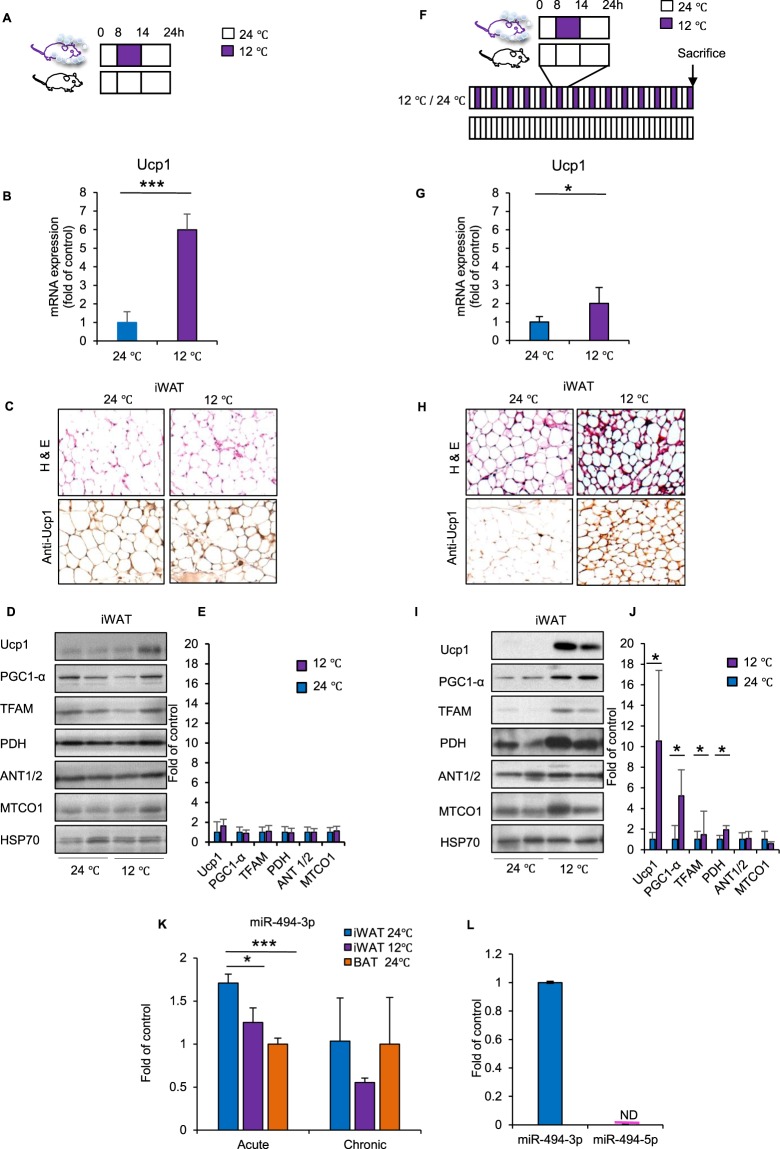
Models for cold exposure in mice. **(A**,**F)** Acute mild cold exposure (**A**) and chronic (**F**) intermittent mild cold exposure protocols. Mice were exposed to 12 °C for 6 h in one day (left, acute protocol) or daily for 14 days (right, chronic protocol) and then sacrificed. Bottom rows of both protocols indicate controls. **(B**,**G**) Ucp1 mRNA expression in iWAT from control and cold exposure groups, normalized by 34B4 mRNA. n = 3–7. **(C**,**H**) Representative images of iWAT. Haematoxylin and eosin staining and Ucp1 immunostaining from control and cold exposure groups (left and right columns, respectively). **(D**,**I**) Immunoblot analysis of the indicated mitochondrial proteins in iWAT from control and cold exposure groups. HSP70 was used as a loading control. Data from two representative mice in each group are shown. **(E**,**J**) Densitometric analysis of mitochondrial proteins in iWAT from acute (**D**) and chronic (**I**) cold exposure groups. Levels were normalized to HSP70 as an internal control. **(K)** Quantification of miR-494-3p expression in iWAT from control and cold exposure groups and BAT from control groups, normalized by U6. n = 3–5. (**L**) Quantification of miR-494-3p and miR-494-5p expression in iWAT from control groups, normalized by U6. n = 8. *p < 0.05, ***p < 0.001. Full-length blots are presented in Supplementary Fig. 5. iWAT: inguinal white adipose tissue; Ucp1: uncoupling protein 1; PGC1-α: PPAR gamma coactivator 1-α; TFAM: transcription factor A mitochondria; PDH: pyruvate dehydrogenase E1-alpha subunit; ANT1/2: adenine nucleotide translocase 1/2; MTCO1: cytochrome c oxidase subunit 1; HSP70: heat shock protein 70; BAT: brown adipose tissue.

To investigate whether miR-494-3p contributes to mitochondrial biogenesis in adipocyte browning *in vivo*, C57BL/6 J mice were subjected to either acute mild cold exposure (6 h at 12 °C, Fig. 1A–E) or chronic intermittent mild cold exposure (6 h/day at 12 °C for 2 weeks, Fig. 1F–J) and adipose tissues were analysed. Control mice were kept at normal temperature (24 °C).

We observed that Ucp1 mRNA expression was significantly increased both in the acute (*p* < 0.001) and chronic cold (*p* < 0.05) exposure groups (Fig. 1B,G), suggesting that mild cold exposure for 6 h in the acute setting was sufficient for the initiation of beige differentiation. Histologically, inguinal white adipose tissue (iWAT) from the acute cold exposure group showed almost no change compared with control iWAT, but the chronic cold group showed high density of haematoxylin and eosin-stained structures along with increased Ucp1 staining in iWAT (Fig. 1C,H). In addition to Ucp1, we examined the levels of key mitochondrial biogenesis regulators, such as PGC1-α and TFAM. PGC1-α and TFAM protein levels were unchanged in the acute cold exposure group compared with control mice (Fig. 1D,E), however we detected significantly increased PGC1-α and TFAM in the intermittent chronic cold exposure group (*p* < 0.05) (Fig. 1I,J). Similarly, pyruvate dehydrogenase (PDH), which glycolysis with the citric acid cycle, was also unchanged in the acute group but increased with chronic cold exposure (Fig. 1D,E,l,J). Adenine nucleotide translocator (ANT1/2) and the mitochondrially encoded cytochrome c oxidase (MTCO1) were unchanged both in the acute and chronic cold exposure groups compared with control conditions (Fig. 1D,E,l,J), These data indicated that acute cold initiated beige differentiation and chronic cold exposure induced beige differentiation, as characterized by increased Ucp1 expression as well as increased mitochondrial biogenesis.

Next, we measured the expression level of miR-494-3p in iWAT and BAT. We found that miR-494-3p expression was lower in BAT compared with iWAT at 24 °C in the acute model (Fig. 1K). Consistent with our previous results in skeletal muscle in response to exercise^15^, miR-494-3p expression was significantly decreased in iWAT upon mild cold exposure compared with control iWAT in the acute model (*p < *0.05). Because two distinct mature microRNAs, mmu-miR-494-3p and mmu-miR-494-5p are generated from pre-miR-494 precursor, we evaluated the miR-494-5p expression in control iWAT, and found that miR-494-5p expression was bellow detection limit by RT-qPCR (Fig. 1L). In the chronic model, miR-494-3p showed a tendency towards decreased expression compared with controls, but the change was not significant.

### Mitochondrial biogenesis during differentiation of 3T3-L1 cells to white and beige adipocytes

**Figure 2:**
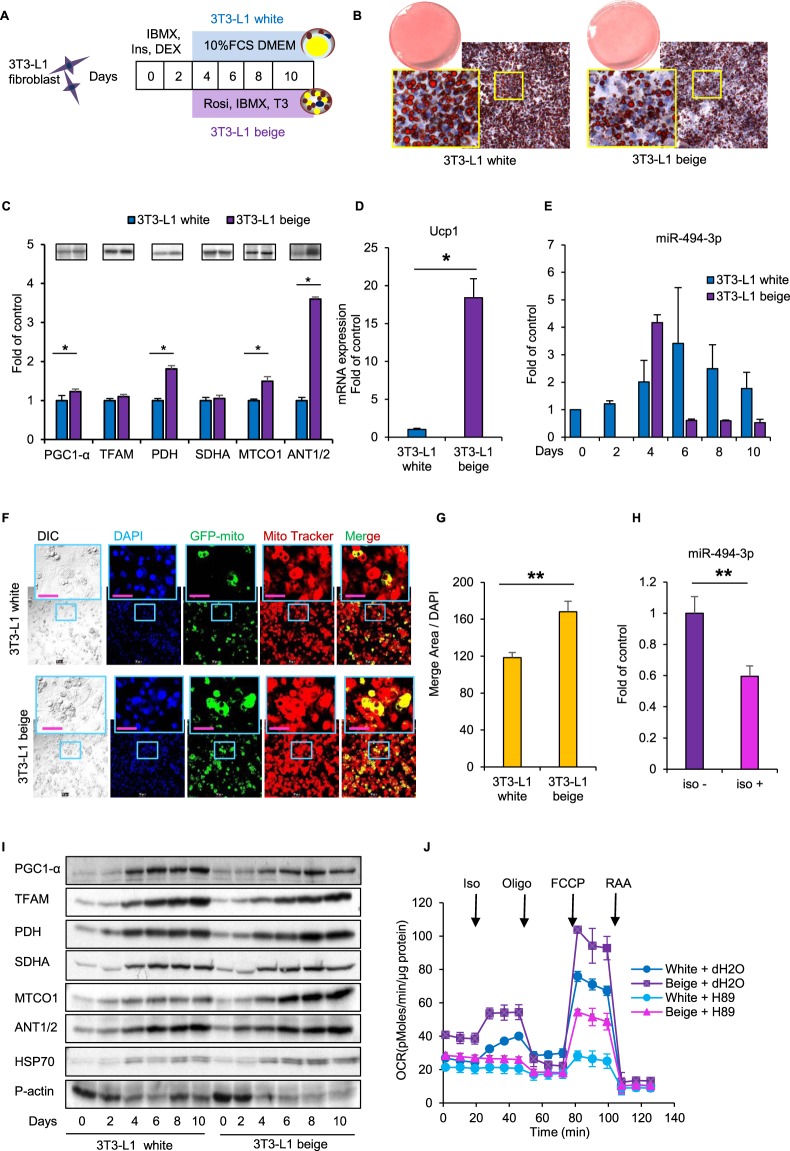
3T3-L1 adipocytes differentiated to matured white and beige type adipocytes. **(A**) Differentiation protocol of 3T3-L1 fibroblasts to white adipocytes and beige adipocytes. Two days after reaching confluence (day 0), cells were cultured for 2 days in medium with Dex (0.25 µM), IBMX (0.5 mM) and Ins (10 µg/ml) and then cultured in regular medium (for white adipocytes) or beige induction medium containing T_3_ (50 nM), Rosi (1 µM), and IBMX (0.5 mM) (for beige-type adipocytes). (**B**) Representative Oil Red O staining images of differentiated 3T3-L1 white and beige adipocytes. Magnification, 100x. **(C)** Densitometric analysis of mitochondrial proteins in 3T3-L1 cells differentiated to matured white and beige type cells harvested at day 8 after isoproterenol (10 µM) treatment for 8 h. HSP70 served as normalization control. Western blots are shown above the columns. n = 6. **(D)** Ucp1 mRNA expression in 3T3-L1 white and beige adipocytes treated with 10 µM isoproterenol on day 8 for 6 h. Levels were normalized with 36B4 mRNA. n = 4. **(E)** miR-494-3p expression during differentiation of 3T3-L1 fibroblasts to matured 3T3-L1 white and beige adipocytes, normalized by U6. n = 3–6. **(F)** 3T3-L1 white and beige adipocytes expressing mito-GFP were treated with iso (10 µM) for 8 h on day 8 and stained with Mitotracker red. Magnification, 200x. Scale bars, 50 µm. **(G)** Quantification of the merge area (yellow) of GFP-mito (green) and Mitotracker-red (red) and adipocyte numbers (quantified by DAPI). n = 4. **(H)** miR-494-3p expression in 3T3-L1 beige cells with or without 10 µM isoproterenol for 6 h, normalized by U6. n = 3. **(I)** Western blot of mitochondrial proteins during differentiation of 3T3-L1 cells to matured white and beige adipocytes. Pan-actin served as loading control. **(J)** Oxygen consumption rate (OCR) in 3T3-L1 white and beige cells pre-treated with H89 (50 µM) for 1 h with the sequential injection of isoproterenol (50 µM), oligomycin (2 µM), FCCP (2 µM), rotenone (R; 0.5 µM) and antimycin A (AA: 0.5 µM) at the indicated times. *p < 0.05, **p < 0.01. Full-length blots are presented in Supplementary Fig. 5. Ucp1: uncoupling protein 1; PGC1-α: PPAR gamma coactivator 1-α; TFAM: transcription factor A mitochondria; PDH: pyruvate dehydrogenase E1-alpha subunit; MTCO1: cytochrome c oxidase subunit 1; SDHA: succinate dehydrogenase subunit A; ANT1/2: adenine nucleotide translocase 1/2; HSP70: heat shock protein 70; P-Actin: pan-actin; iso: isoproterenol; Oligo: oligomycin; RAA: rotenone and antimycin A.

We next investigated the relationship between miR-494-3p and mitochondrial biogenesis in adipocytes. We induced differentiation of 3T3-L1 cells to white and beige adipocytes, as described in Methods (Fig. 2A). Oil Red O staining of 3T3-L1 beige adipocytes revealed a lower degree of lipid droplet accumulation compared with 3T3-L1 white adipocytes (Fig. 2B). Several mitochondrial proteins, such as PGC1-α, PDH, MTCO1 and ANT1/2, were expressed at significantly higher levels in 3T3-L1 beige adipocytes compared with 3T3-L1 white adipocytes at day 8 (*p* < 0.05) (Fig. 2C, Supplementary Fig. S1B). Ucp1 protein levels were undetectable in both 3T3-L1 white and beige adipocytes (Supplementary Fig. S1A); however, Ucp1 mRNA expression was approximately 17-fold higher in 3T3-L1 beige adipocytes compared with white adipocytes (Fig. 2D). We also found that miR-494-3p levels were lower during 3T3-L1 beige differentiation compared with 3T3-L1 white differentiation, miR-494-3p levels peaked at day 6 in 3T3-L1 white adipocytes and day 4 in 3T3-L1 beige adipocytes, followed by a dramatic decrease in both cell types (Fig. 2E). Compared with 3T3-L1 white adipocytes, 3T3-L1 beige adipocytes contained larger amounts of mitochondrial contents, as evaluated by Mitotracker staining and mitochondrial-targeted GFP expression (Fig. 2F,G). Furthermore, β3 adrenergic activation with 10 µM isoproterenol treatment for 8 h resulted in downregulation of miR-494-3p expression in day 8 of 3T3-L1 beige (Fig. 2H). These data indicated that 3T3-L1 beige adipocytes showed higher mitochondrial contents with lower miR-494-3p expression, similar to iWAT in the *in vivo* model. During differentiation of both 3T3-L1 white and 3T3-L1 beige adipocytes, a dramatic increase in PGC1-α and mitochondrial proteins such as TFAM, PDH, succinate dehydrogenase A (SDHA), ANT1/2 and MTCO1 were observed (Fig. 2I).

We then evaluated mitochondrial function by oxygen consumption rate (OCR) analyses in 3T3-L1 white and beige cells (Fig. 2J). We found that the basal OCR was higher in 3T3-L1 beige cells compared with white cells. In addition, the isoproterenol-stimulated increase in OCR was elevated both in 3T3-L1 white and beige adipocytes and blunted by treatment with the PKA inhibitor, indicating the involvement of β3 AR-PKA pathway. In summary, 3T3-L1 beige adipocytes share the signatures of beige adipocytes with the *in vivo* model.

### miR-494-3p overexpression downregulated mitochondrial proteins in 3T3-L1 beige adipocytes

To investigate whether miR-494-3p regulates mitochondrial protein expression in 3T3-L1 white adipocytes, we analysed the effects of overexpression or inhibition of miR-494-3p on the levels of key mitochondrial proteins.

**Figure 3:**
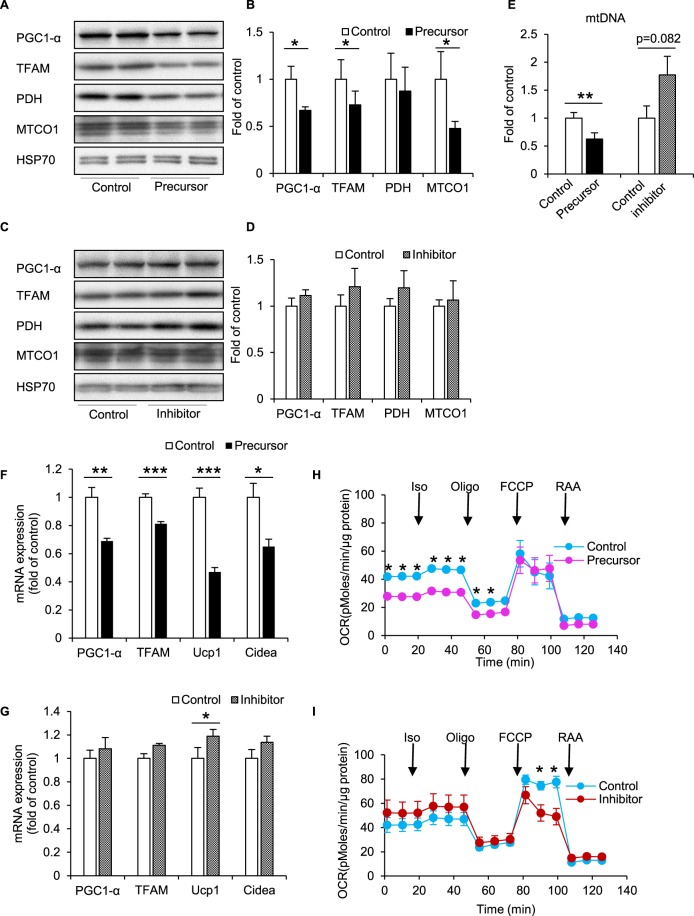
Overexpression of miR-494-3p downregulates the browning and thermogenic gene program in beige adipocytes. **(A**,**C**) Western blotting analysis of 3T3-L1 beige adipocytes transfected with miR-494-3p precursor (**A**), inhibitor (**C**) or controls. Immunoblot shown is representative of three independent experiments. Cells were harvested at day 8 after iso (10 µM) treatment for 8 h. n = 6. **(B**,**D)** Densitometric analysis of proteins from western blot in (**A**) and (**C**). Levels were normalized to HSP70 as an internal control. n = 6 each group. **(E)** mtDNA copy number was analysed in cells harvested at day 8 after iso (10 µM) treatment for 8 h as described in Method (supplementary file 1) **(F**,**G**) RT-qPCR analysis of mitochondrial biogenesis markers. Cells were harvested at day 8 with iso (10 µM) treatment for 8 h. mRNA levels were normalized using 36B4. n = 6. **(H**,**I**) Oxygen consumption rate (OCR) under miR-494-3p overexpression or inhibition in 3T3-L1 beige adipocytes transfected with miR-494-3p precursor, inhibitor or control with 50 µM isoproterenol treatment at 24 min. n = 5. *p < 0.05, **p < 0.01, ***p < 0.001. Full-length blots are presented in Supplementary Fig. 5.

Overexpression of miR-494-3p in 3T3-L1 beige adipocytes resulted in significantly reduced expressions of PGC1-α, TFAM and MTCO1 compared with controls (*p* < 0.05) (Fig. 3A,B, Supplementary Fig. S2A,B) and mtDNA copy number (*p* < 0.05) (Fig. 3E). We confirmed an overexpression of miR-494-3p in 3T3-L1 beige cells by ~1,700 fold compared with controls (Supplementary Fig. S2B). In contrast inhibition of miR-494-3p did not result in significant changes in the examined protein levels (Fig. 3C,D, Supplementary Fig. S2C). However, mtDNA copy number showed a tendency to increase compared with controls (*p* = 0.082) (Fig. 3E). Furthermore, the mRNA levels of mitochondrial genes such as PGC1-α, TFAM, Ucp1 and Cidea genes were significantly downregulated by the overexpression of miR-494-3p (*p* < 0.05) (Table 1) (Fig. 3F), while inhibition of miR-494-3p result in significantly increased Ucp1 mRNA expression (*p* < 0.05) (Fig. 3G).

**Table 1:** Primer sequences.

	Forward primer	Reverse primer
36B4	GCCGTGATGCCCAGGAAGA	CATCTGCTTGGAGCCCACGTT
Cidea	ACTTCCTCGGCTGTCTCAATGTCA	TCAGCAGATTCCTTAACACGGCCT
Ucp1	GGGCATTCAGAGGCAAATCAGCTT	ACACTGCCACACCTCCAGTCATTA
Pgc1a	CATTTGATGCACTGACAGATGGGA	CCATCAGGCATGGACGAA
TFAM	GCAGCCCTGTGGAGGGAGCTA	TCTGCCGGGCCTCCTTCTCC

To test the impact of miR-494-3p on mitochondrial function, we measured OCR in 3T3-L1 beige adipocytes transfected with either the miR-494-3p precursor or miR-494-3p inhibitor. Interestingly, the basal OCR was strongly decreased in the miRNA-494-3p-overexpressing beige cells (Fig. 3H). In contrast, inhibition of miR-494-3p by antisense oligonucleotide in 3T3-L1 beige adipocytes slightly increased the OCR (Fig. 3I).

Next, we analysed the effects of overexpression or inhibition of miR-494-3p in 3T3-L1 white adipocytes. Similar to 3T3-L1 beige adipocytes overexpressing miR-494-3p, PGC1-α and its downstream targets TFAM, PDH and MTCO1 along with mtDNA copy number were significantly downregulated upon miR-494-3p overexpression compared with controls (*p* < 0.05) (Supplementary Fig. S3A–E). Further, inhibition of miR-494-3p by miR-494-3p antisense resulted in significant increases in PGC1-α and PDH protein expression (*p* < 0.05) but had no effect on TFAM and MTCO1 (Supplementary Fig. S3A–E). Together these data revealed that miR-494-3p regulates mitochondrial biogenesis in adipocytes.

### PGC1-α is a potential target of miR-494-3p

**Figure 4:**
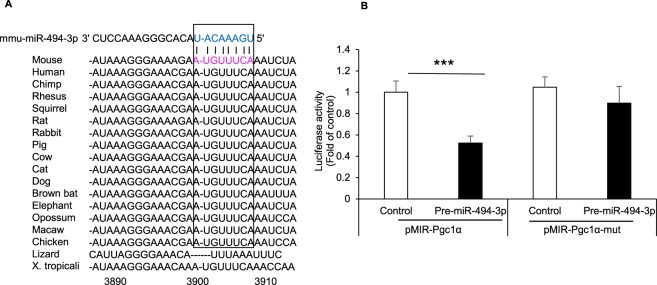
PGC1-α mRNA is a target of miR-494-3p. **(A)** Predicted miR-494-3p target site in the 3′-untranslated region (UTR) of mouse PGC1-α and alignment with sequences in other species. The seed region is enclosed with a square box. **(B)** Luciferase reporter assays. Luciferase reporters containing the PGC1α wild-type or mutant 3′-UTR were transfected along with miR-494-3p precursor or scrambled sequence control into beige adipocytes for 48 h (n = 8). ***p < 0.001.

We next explored the target of miR-494-3p in adipocytes. Our data in 3T3-L1 adipocytes showed lower expression of PGC1-α in response to overexpression of miR-494-3p (Fig. 3B, Supplementary Fig. S3B). This is consistent with the data produced by TargetScan prediction software, which identified PGC1-α as one of the strongest candidate targets of miR-494-3p. We analysed the 3′-UTR of PGC1-α and identified a conserved binding sequence for miR-494-3p across multiple species (Fig. 4A). We thus next performed luciferase assays using a reporter vector containing the 3′-UTR region of PGC1-α inserted downstream of the luciferase coding sequence. The luciferase reporter assays revealed that overexpression of miR-494-3p could repress expression of the luciferase reporter containing the wild-type PGC1-α 3′-UTR but had no impact on the luciferase reporter with the PGC1-α 3′-UTR containing mutations in the putative miR-494-3p binding sequence (Fig. 4B). In addition, we also evaluated the effect of miR-494-5p on PGC1-α 3′-UTR using luciferase assays. We confirmed that miR-494-5p has no impact of the luciferase reporter vector containing the wild PGC1-α 3′-UTR (Supplementary Fig. S4). Together these data suggest that PGC1-α is a target of miR-494-3p and that miR-494-3p targets the PGC1-α gene by binding its 3′-UTR.

### Ucp1 protein was downregulated by miR-494-3p precursor in primary beige cells

To further verify the effect of miR-494-3p in mitochondrial biogenesis in beige cells, we next performed analyses in primary cultured cells, particularly as 3T3-L1 white and beige adipocytes express low levels of Ucp1 protein (Supplementary Fig. S1A).

**Figure 5:**
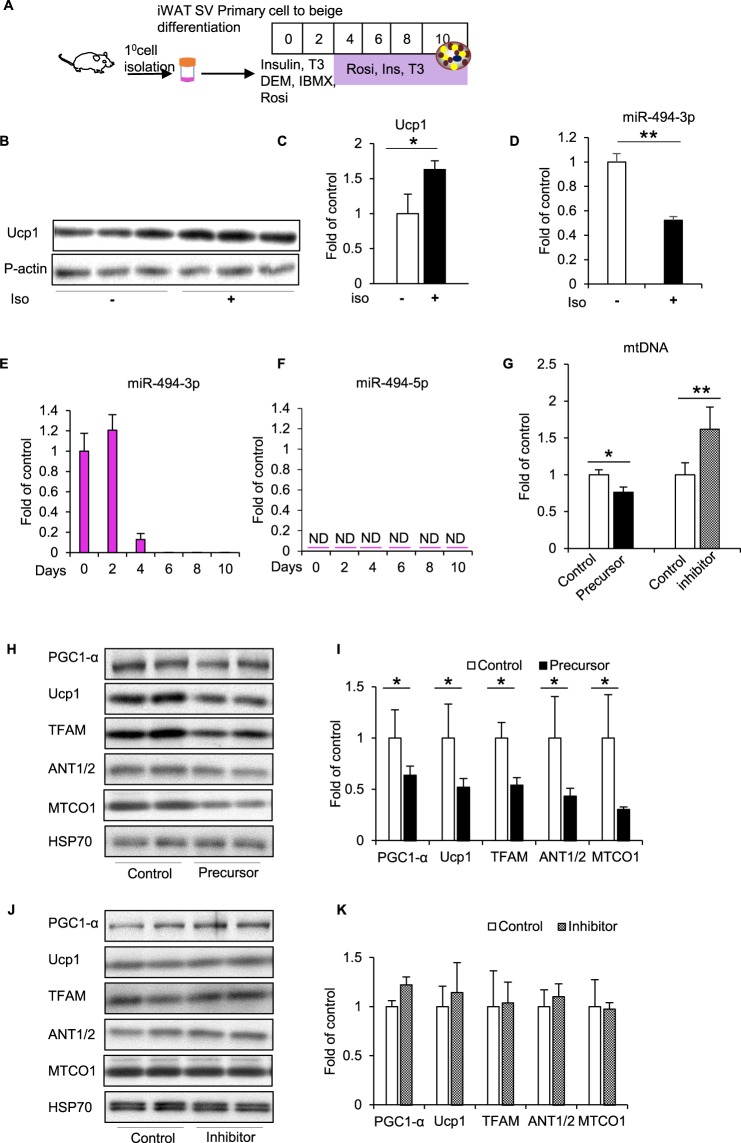
miR-494-3p overexpression or inhibition in primary adipocytes. **(A)** Stromal vascular (SV) cell fractions were isolated from iWAT and differentiated to beige adipocytes using the indicated protocol as described in Methods. Cells were treated with dexamethasone (0.25 µM), IBMX (0.5 mM), insulin (10 µg/mL), T3 (1 nM), and rosiglitazone (0.5 µM). **(B)** Ucp1 expression in beige primary adipocytes with or without isoproterenol treatment (50 µM, 8 h). Results are representative of three independent experiments. Pan-actin served as loading control. **(C)** Densitometric analysis of results from (B). Pan-actin served as normalization control. n = 3. (**D**) miR-494-3p expression in primary beige adipocytes with or without isoproterenol (50 µM, 8 h). Levels were normalized using U6. n = 3. (**E**,**F**) miR-494-3p and miR-494-5p expression during differentiation of primary beige adipocytes from iWAT at different time points. Levels were normalized to U6 as an internal control. n = 4. (**G**) mtDNA copy number was analysed in cells harvested at day 8 after iso (10 µM) treatment for 8 h as described in Method (supplementary file 1). **(H**,**J**) Western blot of in primary adipocytes with miR-494-3p overexpression (**H**) or inhibition (**J**). Cells were harvested at day 8 with iso (10 µM) treatment for 8 h. Results are representative of three independent experiments. n = 6. HSP70 served as an internal control. (**I**,**K**) Densitometric analysis of western blot results (**H**) and (**J**). HSP70 served as control. n = 6. *p < 0.05, **p < 0.01. Full-length blots are presented in Supplementary Fig. 5. Ucp1: uncoupling protein 1; PGC1-α: PPAR gamma coactivator 1-α; TFAM: transcription factor A mitochondria; ANT1/2: adenine nucleotide translocase 1/2; MTCO1: cytochrome c oxidase subunit 1; HSP70: heat shock protein 70; iso: isoproterenol.

Isolated stromal vascular (SV) cells of iWAT from 8-week-old C57BL/6 mice were differentiated into matured beige adipocytes, as described in Methods (Fig. 5A). Ucp1 protein expression was detected at day 8 by immunoblotting and was enhanced by isoproterenol treatment (Fig. 5B,C). We also detected a significant decrease in miR-494-3p expression in response to isoproterenol treatment (*p* < 0.01) (Fig. 5D). We measured the expression level of miR-494-3p and miR-494-5p in iWAT during differentiation at different time points. We found that miR-494-3p expression was decreased during maturation from day 4 to day 10 (Fig. 5E). However, miR-494-5p expression was below the detection limit by RT-qPCR, indicating that miR-494-5p was not expressed in iWAT (Fig. 5F). mtDNA copy number was significantly decreased by miR-494-3p overexpression and significantly increased by miR-494-3p inhibition (*p* < 0.05) (Fig. 5G). Furthermore, overexpression of miR-494-3p in primary beige adipocytes significantly reduced Ucp1 protein levels (*p* < 0.05) (Fig. 5H,I). Moreover, PGC1-α, TFAM, ANT1/2, and MTCO1 protein expressions were also significantly downregulated in primary beige cells with miR-494-3p overexpression (*p* < 0.05), similar to the effects observed in 3T3-L1 adipocytes.

**Figure 6:**
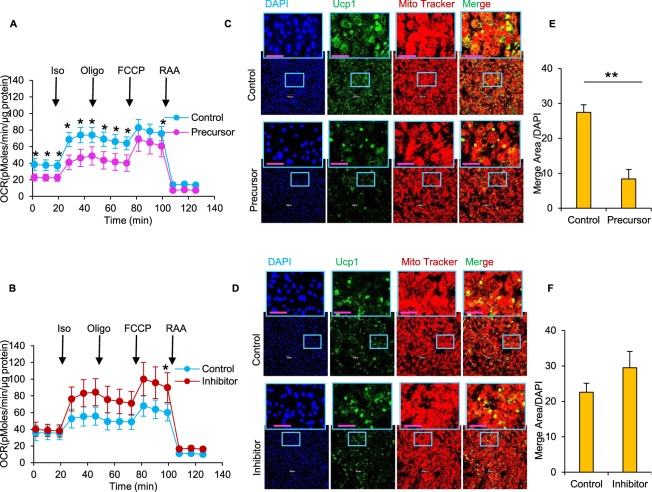
Overexpression of miR-494-3p reduces oxygen consumption rate and Ucp1 expression in primary adipocytes. **(A**,**B**) Oxygen consumption rate (OCR) in primary beige adipocytes with miR-494-3p overexpression (**A**) or inhibition (**B**) treated with isoproterenol (50 µM), oligomycin (2 µM), FCCP (2 µM), rotenone (R; 0.5 µM) and antimycin A (AA; 0.5 µM) as indicated. n = 5. (**C**,**D**) Representative images of UCP1 immunofluorescence staining (green) and Mitotracker red staining (red) in primary differentiated beige adipocytes with miR-494-3p overexpression (**C**) or inhibition (**D**). Cells were harvested at day 8 with iso (10 µM) treatment for 8 h. (**E**,**F**) Quantification of the merge area (yellow) of GFP-mito (green) and mito-red (red) and number of adipocytes (indicated by DAPI) using ImageJ in cells with miR-494-3p overexpression (**E**) or inhibition (**F**). n = 8–10. *p < 0.05, **p < 0.01. Ucp1: uncoupling protein 1; iso: isoproterenol; Oligo: oligomycin; RAA: rotenone and antimycin A.

We also measured OCR in primary adipocytes to examine the role of miR-494-3p in mitochondrial function. During miR-494-3p overexpression, we detected a significant decrease of OCR under basal, isoproterenol-stimulated and oligomycin treatment conditions in miR-494-3p overexpressing adipocytes compared with controls (Fig. 6A). However, inhibition of miR-494-3p had no effects on mitochondrial protein expression (Fig. 5J,K), probably because of low endogenous expression in the differentiated cells. Treatment of primary beige cells with the miR-494-3p inhibitor had little effect on OCR in primary beige adipocytes (Fig. 6B), similar to our observations in 3T3-L1 beige adipocytes (Fig. 3I).

To confirm whether the miR-494-3p precursor exhibits cellular effects on thermogenic stimulation and mitochondrial biogenesis, we conducted live staining of primary beige adipocytes using Mitotracker red and immunofluorescence against Ucp1. Overexpression of miR-494-3p decreased Mitotracker red staining and Ucp1 immunostaining compared with the control group (Fig. 6C,E), while inhibition of miR-494-3p had little effect (Fig. 6D,F). The effect of miR-494-3p in mitochondrial biogenesis in primary beige cells was consistent with the results of 3T3-L1 beige adipocytes.

## Discussion

**Figure 7:**
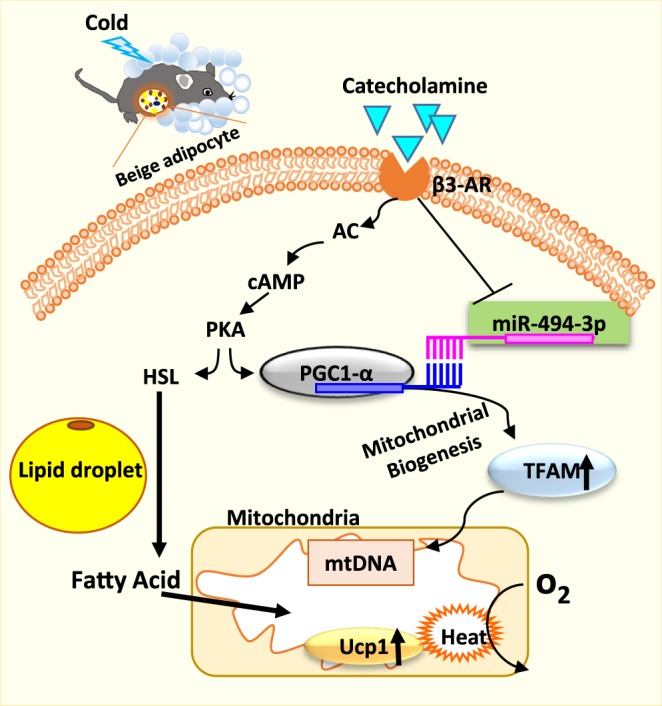
A proposed model for the roles of miR-494-3p in mitochondrial biogenesis and thermogenesis in beige adipocytes. In this model, cold/β_3_-AR adrenergic stimulation causes a decrease in miR-494-3p expression in beige adipocytes, thereby inducing mitochondrial biogenesis and thermogenesis. Catecholamine binds to β_3_-AR to activate the cAMP pathway and inhibit miR-494-3p expression, reducing the inhibitory activity of miR-494-3p on PGC1-α gene expression. β_3_-AR: β_3-_ adrenergic receptor; AC: adenylate cyclase; cAMP: cyclic AMP; PKA: protein kinase A; HSL: hormone sensitive lipase; TFAM: mitochondrial transcription factor A; mtDNA: mitochondrial DNA; UCP1: uncoupling protein 1.

The present study was performed to clarify the role of miR-494-3p in the differentiation of beige adipocytes. This study has revealed three important findings (Fig. 7). First, the expression of miR-494-3p was reduced in iWAT by cold exposure *in vivo* and was also reduced in response to beige induction, with a corresponding increase of mitochondrial proteins. Second, the expression of miR-494-3p was decreased by β3 adrenergic stimulation in beige adipocytes. Third, overexpression of miR-494-3p reduced PGC1-α mRNA and protein levels in adipocytes and attenuated mitochondrial biogenesis and oxygen consumption. These findings demonstrate that miR-494-3p directly inhibits the expression of PGC1-α and subsequently mitochondrial biogenesis in adipose tissue. The decreased miR-494-3p expression during adipocyte differentiation removes its inhibitory effect, leading to stimulation of Ucp1 expression and mitochondrial biogenesis.

Our results showed that expression of miR-494-3p was reduced in iWAT by acute mild cold exposure and showed a tendency for reduction by chronic intermittent cold exposure *in vivo* (Fig. 1K). We also observed reduced miR-494-3p expression in response to beige induction (Fig. 5E). These findings are consistent with our previous report, in which acute exercise reduced the expression of miR-494-3p in skeletal muscle in mice^15^, and reports from others that showed reduced miR-494-3p expression in muscle after exercise^17,18^. It is unclear why chronic intermittent cold exposure showed large variation in miR-494-3p. It is possible that the intermittent and mild (12 °C) cold exposure may methodologically result in the large variations of expression. In fact, we found a similar phenomenon in skeletal muscle from mice after intermittent 2 h swimming periods over 2 weeks. Concerning temperature, enhanced mitochondrial protein expression in response to cold exposure was consistent with a previous report that showed that 14 weeks of intermittent 4 °C treatment increased the protein expression of Ucp1 and PGC1-α in iWAT^19,20^.

We also found that the expression of miR-494-3p was decreased by β3 adrenergic stimulation in beige adipocytes (Figs 2H, 5D). Cold exposure is a known stimulus for both beige formation and sympathetic nerve activation. The β3-AR knock-out mice showed almost no Ucp1 induction after acute and chronic cold exposure, indicating that β3-AR activation plays an essential role in beige differentiation^21^. Exercise is known to increase sympathetic nerve tone and mitochondrial biogenesis, which was shown by increased PGC1-α^22^. The mechanism of adaptation of skeletal muscle to exercise may be similar to the mechanism we observed in the adaptation in adipose tissue against cold in this study. To confirm the impact on mitochondrial biogenesis by miR-494-3p, we performed further investigations.

Our results showed that miR-494-3p reduced the protein expression of PGC1-α in adipocytes and attenuated mitochondrial biogenesis and oxygen consumption. We used computational miRNA target prediction algorithms to identify PGC1-α as one of the target genes for miR-494-3p. We confirmed the miR-494-3p binding site in the 3′-UTR of PGC1-α (Fig. 4B), a master regulator of mitochondrial biogenesis and function^7^. This binding sequence is evolutionally conserved in mammals including human and mouse and birds, but not in lizard and *Xenopus tropicalis*, suggesting that homoeothermic animals share features to adapt to cold environments. We also showed that miR-494-3p overexpression in beige adipocytes led to downregulation of mRNA and protein expression of PGC-1α along with reduced expression of its downstream genes. A previous report showed that miR-494-3p is expressed in mitochondria and regulates the mtDNA genome^14^. Our group, as well as others, previously reported that TFAM, FoxJ3, and CREB1 are targets of miR-494-3p^15,23^, suggesting that miR-494-3p serves as a fine-tuner of mitochondrial biogenesis.

miR-494-3p decreased oxygen consumption in both Ucp1-dependent and Ucp1-independent manners. Although 3T3-L1 beige adipocytes may express low levels of Ucp1 protein, we found that β3-adrengenic stimulated an increase in the OCR. Consistent with our results, other researchers reported that OCR can be elevated in a Ucp1-independent manner through ANT1/2^24,25^. In our study, we detected high levels of ANT1/2 that were increased by cold exposure *in vivo* or during 3T3-L1 beige differentiation. Differences in OCR between 3T3-L1 white and beige adipocytes could be reasonably explained by the observed differences in ANT1/2 between these cells. This question requires further investigation.

Recent studies have revealed the possibility that induction of beige differentiation by activating the thermogenic gene program in beige adipocytes in rodents and adult humans could increase whole-body energy expenditure, and therefore could protect against obesity and diabetes^26,27^. Thus, many genes and pathways that regulate beige adipocyte biology may provide a variety of promising therapeutic targets for metabolic disease^28^.

There are two main strengths of this study. First, to the best of our knowledge, a broad examination of the expression trends of mitochondrial proteins in multiple beige models has never been conducted until now. Notably, an association between PGC1-α and miR-494-3p expression was consistently observed among these models. Second, this paper suggested the importance of a Ucp1-independent proton leak in the adaptation for cold environment through beige differentiation.

This study had some limitations. First, many direct target genes of miR-494-3p other than PGC1-α may exist, and these additional targets may explain the inconsistencies in the expressions of several mitochondrial genes in our study. Our group previously reported that miR-494-3p expression was reduced after exercise and mitochondrial biogenesis was directly supressed through TFAM and Foxj3 in the skeletal muscle. Our preliminary experiments revealed no significant differences in TFAM-3′-UTR luciferase activity by overexpression of miR-494-3p in 3T3-L1 beige cells, suggesting that targets may vary in different tissues (Supplementary Fig. S4B)^15^. Second, the miR-494-3p inhibitor had little effect on beige adipogenesis. Because the efficacy of miR-494-3p antisense was higher in 3T3-L1 white cells compared with 3T3-L1 beige or primary beige cells, we speculated that the endogenous expression of miR-494-3p is an important factor for loss of function experiments. To more clearly examine the function of miR-494-3p in beige adipocytes, we have initiated the construction of miR-494 flox mice using the CRISPR-Cas9 system. Further experiments in miR-494 knockout mice may help to elucidate the role of miR-494 in thermogenesis *in vivo*.

Together, our findings demonstrated that decreased miR-494-3p levels during beige differentiation stimulated Ucp1 and mitochondrial biogenesis through reducing the inhibitory activity of miR-494-3p on PGC1-α gene expression.

## Methods

The following reagents were purchased from Sigma (St. Louis, MO, USA): dexamethasone (Dex, D4902), 3-isobutyl-1-methylxanthine (IBMX, 17018), insulin (Ins, 16634), triiodothyronine (T_3_, T5516), rosiglitazone (Rosi, R2408) and isoproterenol (Iso, 16504). Dulbecco’s Modified Eagle’s Medium (DMEM) and foetal bovine serum (FBS) were obtained from Life Technologies (Grand Island, NY, USA).

### Animals and experimental design

This study was approved by the Animal Care and Use Committee of Shiga University of Medical Science. Male C57BL/6 J mice (8-week-old) were obtained from Charles River Japan and maintained on a chow diet with ad libitum access to water. Cold exposure experiments were conducted according to previous studies^29^, with little modification. Mice were randomly assigned (n = 5/group) into a mild cold group (12 °C) or control group (24 °C). Cold exposure (12 °C) was conducted using a cool cube mounted in a small fridge (HP-500, Hoco, China). In the acute cold exposure experiments, mice were treated with cold exposure for 6 h (9:00 am to 3:00 pm). In the chronic cold exposure experiments, mice were treated with cold exposure for 6 h per day (9:00 am to 3:00 pm) for two weeks. In both experiments, the control groups (24 °C) did not receive cold exposure. After 24 h (acute cold experiments) or two weeks (chronic cold experiments), mice were anesthetized with sevoflurane and the adipose tissues were dissected. The tissues were immediately frozen in liquid nitrogen and stored at −80 °C.

### Immunohistochemical staining

For histological examination, a portion of the adipose tissue was fixed with 3.7% neutrally buffered formaldehyde and embedded in paraffin. The paraffin-embedded sections were deparaffinized and incubated for 30 min with 0.3% H_2_O_2_ in methanol to block endogenous peroxidase. Endogenous avidin and biotin were blocked using an Avidin-Biotin Blocking kit (DAKO, Carpentaria, CA, USA), as described previously^30^. Tissue sections were incubated overnight at 4 °C with primary antibody against Ucp1 (U6382, 1:50; Sigma), followed by 1 h at room temperature with peroxidase-conjugated anti-rabbit IgG secondary antibody (Histofine Simple Stain Max-PO (R); 1:1000; Nichirei, Tokyo, Japan). Immune complexes were visualized using the peroxidase stain DAB kit (brown stain; Nacalai Tesque, Kyoto, Japan) according to the manufacturer’s instructions. Confocal microscopic imaging was used to visualize results.

### Western blotting

Proteins were separated by SDS-PAGE and transferred to polyvinylidene fluoride membranes. Immunoblotting was performed using the following primary antibodies diluted in 5% milk: rabbit anti-mtTFA (1:1000; LS-C30495; LifeSpan BioSciences, Seattle, WA, USA), mouse anti-succinate dehydrogenase complex, subunit A (SDHA; 1:2000; Ab14715; from Abcam, Cambridge, UK), mouse anti-Ucp1 (1:1000; ab23841), anti-MTCO1 (1:1000; ab14705), rabbit anti-PGC1-α (1:1000; ab54482), mouse anti-heat shock protein (HSP70; 1:2000; ab2787), anti-PKA alpha/beta/gamma (phosphorT197) antibody (pPKA; 1:1000; ab75991), rabbit anti-adenine nucleotide translocator (ANT1/2; 1:1000; SC-9299; from Santa Cruz Biotechnology, Santa Cruz, CA, USA), goat anti-pan actin (1:2000; SC-1616), rabbit anti-hormone sensitive lipase (HSL; 1:2000; #41078; Cell Signalling Technology, Beverly, MA, USA), anti-pyruvate dehydrogenase (PDH; 1:3000; Novus Biologicals, Cambridge, UK), and anti-ATGL (1:1000; #2138 s; Cell Signalling Technology). We used the following secondary antibodies diluted at 1:1000 in 5% milk: goat anti-mouse IgG-HRP (NA931V), goat anti-rabbit IgG-HRP (NA934V; both from GE Healthcare, Amersham, UK), and donkey anti-goat IgG-HRP (H2113; Santa Cruz). Protein bands were visualized using a chemiluminescence detection reagent (PerkinElmer, Waltham, MA, USA). The protein band intensities were quantified using Image J software.

### miRNA quantification

TaqMan miRNA assays (Life Technologies) were used for quantifying mmu-miR-494-3p (ID: 002365) and mmu-miR-494-5p (ID: 463045_mat) using RT-qPCR according to the manufacturer’s instructions. U6 small RNA (ID: 001973) was used as an endogenous control for miRNA expression analysis. Ct values were determined at 0.1∆Rn threshold level after automatic baseline calibration. Expression levels of individual miRNAs were determined by the ∆∆Ct approach relative to the average Ct of normalization controls (U6). Expression changes of paired samples were determined by the ∆∆Ct approach^31^.

### Cell culture

Mouse embryo 3T3-L1 pre-adipocytes were provided by Dr. J. M. Olefsky (University of California, San Diego, CA) and cultured as previously reported^32^. Briefly, cells were maintained in growth medium containing DMEM (25 mM glucose) supplemented with 10% FBS at 37 °C in 10% CO_2_ until confluent. Two days after reaching confluence (day 0), the cells were cultured for 2 days in differentiation medium, DMEM containing 10% fetal calf serum (FCS), Dex (0.25 µM), IBMX (0.5 mM) and Ins (10 µg/ml), followed by culture in DMEM containing 10% FCS for white adipocytes and beige induction medium containing T_3_ (50 nM), Rosi (1 µM), and IBMX (0.5 mM) for beige type adipocytes (Fig. 2A). For β3 adrenergic stimulation, 10 µM of isoproterenol (iso) was added to the medium for 8 h on day 8^32,33^.

### MitoTracker staining and confocal microscopy

MitoTracker Red CMXRos (CAT# M-7512; Invitrogen Molecular Probes, Inc., Eugene, OR, USA), a mitochondria-specific cationic fluorescent dye, was used to label mitochondria. The 3T3-L1 cells grown on BD Flacon culture slides were transfected with a mitochondrial-targeted GFP expression vector (a gift from Dr. Yasuo Mori) and stained with 250 nmol/L MitoTracker in serum-free DMEM for 15 min at 37 °C according to the manufacturer’s instructions. An Olympus FLUOVIEW FV1000 confocal laser scanning microscope oil-immersion objective lens was used to characterize the optical properties of these samples.

### Oxygen consumption rate (OCR)

Premature 3T3-L1 adipocytes were seeded into XFe24 Microplates (Seahorse Bioscience, North Billerica, MA, USA) at a density of 25,000 cells/well. Cells were grown to confluence and differentiated into mature adipocytes following the protocol as described above. Cells were washed three times and incubated in non-buffered DMEM supplemented with 25 mM glucose and 1 mM sodium pyruvate for 1 h. The compounds used to determine OCR included 2 μM oligomycin, 0.5 μM carbonyl cyanide-p-trifluoromethoxyphenylhydrazone (FCCP), 2 μM antimycin A and 0.5 μM rotenone. The bioenergetics profile provided by the Seahorse Cell Mito Stress Test kit was determined by adjusting the values to the antimycin/rotenone treatment, which permits a more focused assay on the proton leakage. The OCR value were normalized with protein concentration at the end of the experiment.

### Cell transfection

Differentiated 3T3-L1 adipocytes and primary cells were transfected with mmu-miR-494-3p precursor (#AM17100, ID: PM12409), mmu-miR-494-3p inhibitor (#AM17000, ID: AM12409), mmu-miR-494-5p precursor (#AM17100, ID: PM19833) or mmu-miR-494-5p inhibitor (#AM17000, ID: AM19833) all from Life Technologies (Carlsbad, CA, USA), using Lipofectamine RNAiMAX (Invitrogen, Carlsbad, CA, USA) according to the protocol provided by the manufacturer. Cells were transfected at day 5 after white adipocyte induction and then assayed at day 8. For the control groups, cells were transfected with the precursor control, Pre-miR Negative Control #1 (#AM17110) or the inhibitor control, anti-miR Negative control #1 (#AM17010), both from Ambion by Life Technologies (Carlsbad, CA, USA). To monitor transfection efficiency, 3T3-L1 beige cells were first transfected with FAM-labelled anti-miR negative control (ID: AM17012; Applied Biosystems, Carlsbad, CA, USA) and FAM-labelled pre-miR negative control #1 (ID: AM17110). Transfected cells were examined by fluorescence microscopy using a GFP filter.

### Luciferase reporter assay

Dual-reporter expression clones of murine wild-type and mutated PGC1-α 3′-UTR were obtained from Genecopoeia (Rockville, MD, USA). The sequences are shown in Fig. 4A. The mutant PGC1-α 3′-UTR reporter was created by mutating the seed regions of the predicted mmu-miR-494-3p site (AUGUUUC to CUGCUCC). The 3′-UTR sequences were inserted in the pEZX-MT06 vector (Genecopoeia) downstream of a firefly luciferase reporter gene driven by an SV40 enhancer. The Renilla Luciferase reporter was used as an internal control for transfection efficiency. The plasmids were transfected into beige-type 3T3-L1 cells using DharmaFECT Duo (Cat# T-2010-03; Dharmacon, Lafayette, CO, USA) according to the manufacturer’s instructions. Dual luciferase activity was measured using the Luc-Pair miR luciferase assay (Genecopoeia) according to the manufacturer’s instructions.

### Primary beige adipocyte differentiation from stromal vascular culture

The iWAT fat pad (eight fat pads) was dissected from 8-week-old C57BL/6 mice. The primary stromal-vascular fraction (SVF) was differentiated into beige adipocytes, as previously described^34^. Briefly, tissues were minced and digested with 1.5 U/ml collagenase D (1108874103, Roche, Mannheim, Germany) in 10 mM CaCl_2_ and 2.4 U/ml dispase II (04942078001, Roche) for 40–50 min while shaking at 37 °C. Digestion was stopped by adding complete DMEM/F12 containing 10% FCS and penicillin/streptomycin (SV culture medium). Cells were collected by centrifugation at 700 × *g* for 10 min, resuspended, and strained through a 70 µM cell strainer (BD Biosciences, Tokyo, Japan). Cells were further filtered through a 40 µm cell strainer to remove clumps and large adipocytes. SV cells were re-suspended in SV culture medium and plated onto 6-well collagen-coated dishes. At confluency (day 0), cells were exposed to a differentiation medium including Dex (0.25 µM), IBMX (0.5 mM), Ins (10 µg/mL), T3 (1 nM), and Rosi (0.5 µM) in SV culture medium. At 48 h after induction, the cells were maintained in SV culture medium containing Ins (5 mg/mL) and Rosi (1 µM) for 6 days. Cells were fully differentiated on day 8.

### Immunofluorescence staining

SV cells were plated onto glass chamber cell culture slides and differentiated into beige cells as described above. On day 8, cells were washed with PBS and fixed by incubation with 10% formalin for 20 min at room temperature. Cells were blocked in 2% BSA in PBS and incubated overnight at 4 °C with primary antibody against Ucp1 (1:50; U6382; Sigma-Aldrich) and then incubated with Alexa Fluor 488-conjugated secondary antibody (2 mg/ml). Culture slides were mounted and imaged on an Olympus FLUOVIEW FV1000 confocal laser scanning microscope. An oil-immersion objective lens was used to characterize the optical properties of these samples. The merge area and the adipocyte numbers in the total area were measured from randomly selected fields using semi-automated morphometry (ImageJ, plugin Adipocytes Tool; National Institutes of Health, Bethesda, MD, USA; http://imagej.nih.gov/ij/).

### Statistical analyses

Results are expressed as mean ± SEM. Student’s t test was used to evaluate differences between two groups. One-way ANOVA and a subsequent post hoc Tukey test were used to determine the significance of differences where multiple comparisons were required. *P* < 0.05 was considered statistically significant.

## Electronic supplementary material

Supplementary File 1

Supplementary File 2

## Data Availability

All data generated or analysed during this study are included in this published article and its Supplementary Information files.
