# Supplementary material for: MiR-494-3p regulates mitochondrial biogenesis and thermogenesis through PGC1-α signalling in beige adipocytes
Source: Sci Rep. 2018 Oct 10;8:15096. doi: 10.1038/s41598-018-33438-3 (PMC6180067; doi:10.1038/s41598-018-33438-3)
Supplement: Supplementary file 1 [file 41598_2018_33438_MOESM1_ESM.pdf]

SUPPLEMENTAL INFORMATION

MiR-494-3p regulates mitochondrial biogenesis and thermogenesis through PGC1-α signalling in beige adipocytes

Mengistu Lemecha[1], Katsutaro Morino[1]*, Takeshi Imamura[2], Hirotaka Iwasaki[3], Natsuko Ohashi[1], Shogo Ida[1], Daisuke Sato[1], Osamu Sekine[1], Satoshi Ugi[1] and Hiroshi Maegawa[1]

[1]Division of Endocrinology and Metabolism, Department of Medicine, Shiga University of Medical Science, Japan

[2]Division of Molecular Pharmacology, Faculty of Medicine, Tottori University, Japan

[3]Division of Pharmacology, Shiga University of Medical Science, Japan

*Address correspondence to: Katsutaro Morino, MD, PhD

Shiga University of Medical Science

Tsukinowa, Seta, Otsu, Shiga, 520-2192

Phone: +81-77-548-2222

Fax: +81-77-543-3858

E-mail: morino@belle.shiga-med.ac.jp

SUPPLEMENTAL METHODS

Oil O Red staining

Cell cultures were washed with 10% PBS and then fixed for 30 min with 10% formalin in PBS (formalin 37%; Cat# 16223-55, Nacalai Tesque). Cells were washed with sterile double distilled water and subsequently washed with 60% isopropanol (Cat# 29112-95, Nacalai Tesque) for 2 min. Cells were stained with a filtered 0.35% Oil Red O (Cat# O-0625, Sigma) solution in 60% isopropanol for 10 min at room temperature. Cells were washed with sterile double distilled water then stained with haematoxylin for 10 sec and images of stained cells were taken by microscopy using 10x optical zoom.

Nucleic acid isolation

RNA was isolated using a miRvana PARIS kit (Life Technologies) according to the manufacturer's instructions. Small RNAs were concentrated for quantification of mature miRNA.

Real-time qPCR analysis

The qPCR reaction setup and plate preparation were standardized and carried out according to standard operating protocols as described previously[1]. Briefly, single-stranded cDNA was synthesized from 1.5 μg of total RNA using the Prime Script RT Reagent Kit (Takara Bio, Shiga, Japan), and endogenous genomic DNA was degraded by DNase I (Life Technologies, CA, USA). RT-qPCR experiments were carried out with SYBR Green PCR master mix (Life Technologies) using the ABI 7500 Fast Real-Time PCR System (Applied Biosystems, Foster City, CA, USA). All quantitative data were normalized against the expression levels of 36B4. RT-qPCR conditions were 95°C for 10 min, followed by 40 cycles of 95°C for 15 s and 60°C for 1 min. The amplification efficiency for each primer pair was determined by a qPCR assay using duplicates of a 10-fold dilution series (1:10, 1:100, 1:1000, 1:10.000, 1:100.000) of cDNA as a template. The mean Ct values for each serial dilution were plotted against the logarithm of the cDNA dilution factor. The amplification efficiency for each primer pair was calculated by standard curve methods. The Ct value for all our experiments are available in the separated file (Supplementary file 2). Ct value >35 were considered bellow detection limit in our study.

Quantitative PCR for mtDNA content

MtDNA content was measured as previously described [1]. Briefly, DNA primers were designed to detect cytochrome oxidase 2 (COX2) and uncoupling protein 2 (UCP2) for mitochondrial DNA (mtDNA) and nucleic DNA, respectively (COX2 forward: 5′-TTTTCAGGCTTCACCC TAGATGA-3′, COX2 reverse: 5′-GAAGAATGTTATGTTATGTTTACTCCTA-3′, UCP2 forward: 5′ -GCGACCAGCCCATTGTAGA-3′, UCP2 reverse: 5′-GCGTTCTGGGTAC CATCCTAAC-3′). The ratio of COX2 to UCP2 within the samples was used to calculate the mtDNA content.

Supplementary Figure 1

A

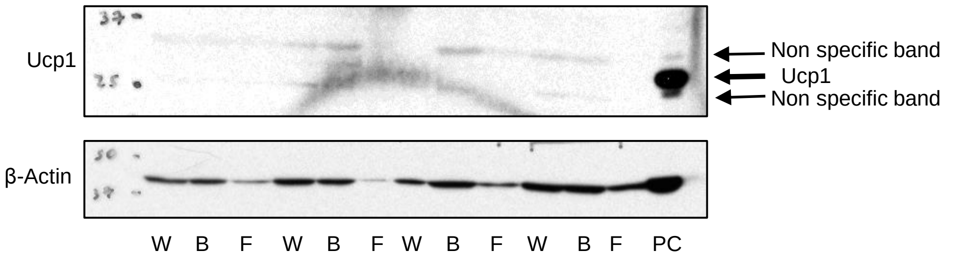

B

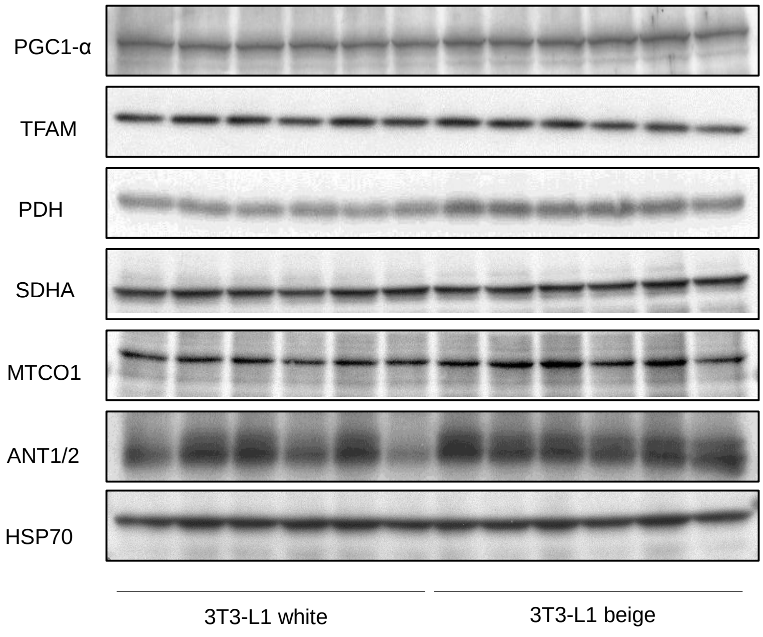

Supplementary Figure 1. Immunoblotting analysis against indicated antibodies.

A: Ucp1 protein expression was not detected in in 3T3-L1 fibroblast (F), 3T3-L1 white adipocyte (W), 3T3-L1 beige adipocyte (B) compared with mouse brown adipose tissue used as a positive control (PC). n=3. **B:** Western blotting of mitochondrial proteins in 3T3L-1 differentiated to matured white and beige adipocytes at day 8. Iso treated for 8 h on day 8. n=4.

Supplementary Figure 2

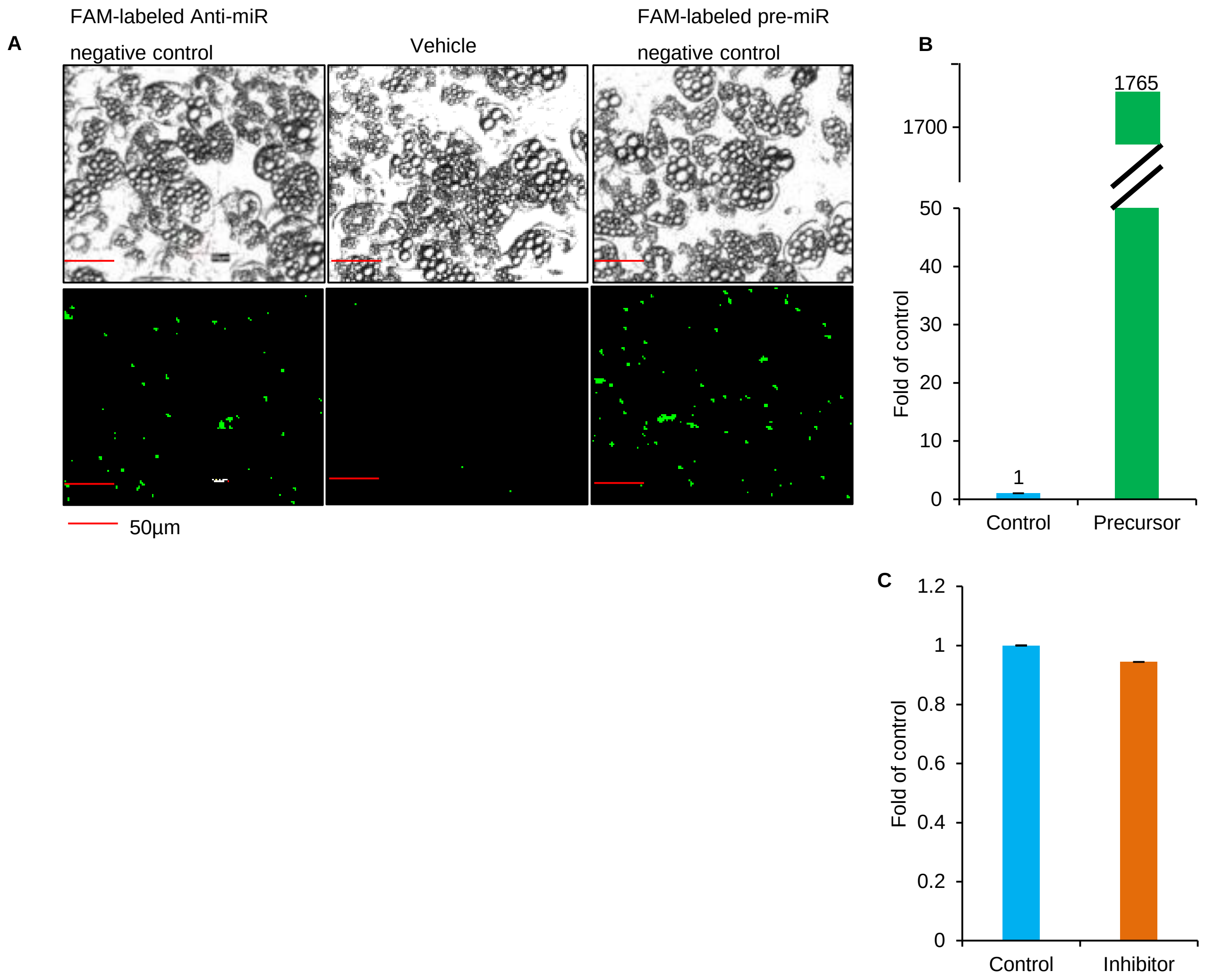

Supplementary Figure 2. miR-494-3p transfection efficiency and level.

(A) Transfection efficiency of precursor and inhibitor of 3T3-L1 beige cells were determined with FAM-labeled Anti-miR negative control and FAM-labeled pre-miR negative control. The transfected cells were then examined by Olympus FLUOVIEW FV1000 confocal laser scanning microscope using GFP filter. n=4. **(B, C)** Expression level of miR-494-3p on day 7 after transfection of precursor or inhibitor compared to vehicle wells. n=4.

Supplementary Figure 3

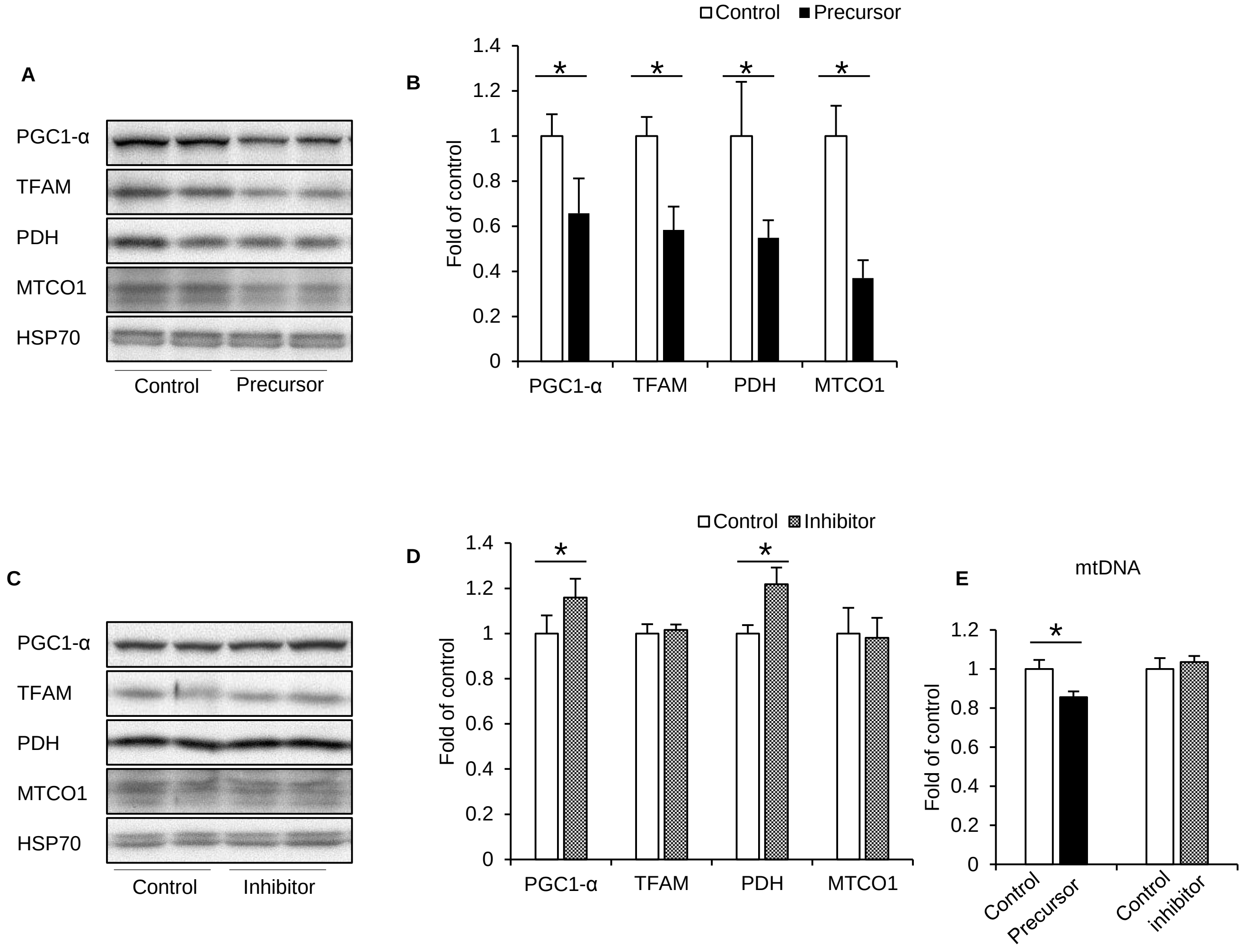

Supplementary Figure 3. miR-494-3p inhibits mitochondrial biogenesis in 3T3-L1 white adipocytes.

(A, C) Western blotting in 3T3-L1 white adipocytes transfected with miR-494-3p precursor or inhibitor. Immunoblot shown is representative of three independent experiments. On day 8 treated with iso (10µM) for 8 h. n =6. **(B, D)** Densitometric analysis of proteins from western blot in (A) and (C). Levels were normalized to HSP70 as an internal control. n=4. **(E)** The expression of a mitochondria genome coding gene [mitochondrial (mt) cytochrome oxidase 2 (COX2)] and a nuclear genome coding gene [g-uncoupling protein 2 (UCP2]) were analyzed using specific primers. Iso treated for 8 h on day 8. Values represent the ratio of the amount of mtCOX2 to gUCP2). *p < 0.05.

Supplementary Figure 4

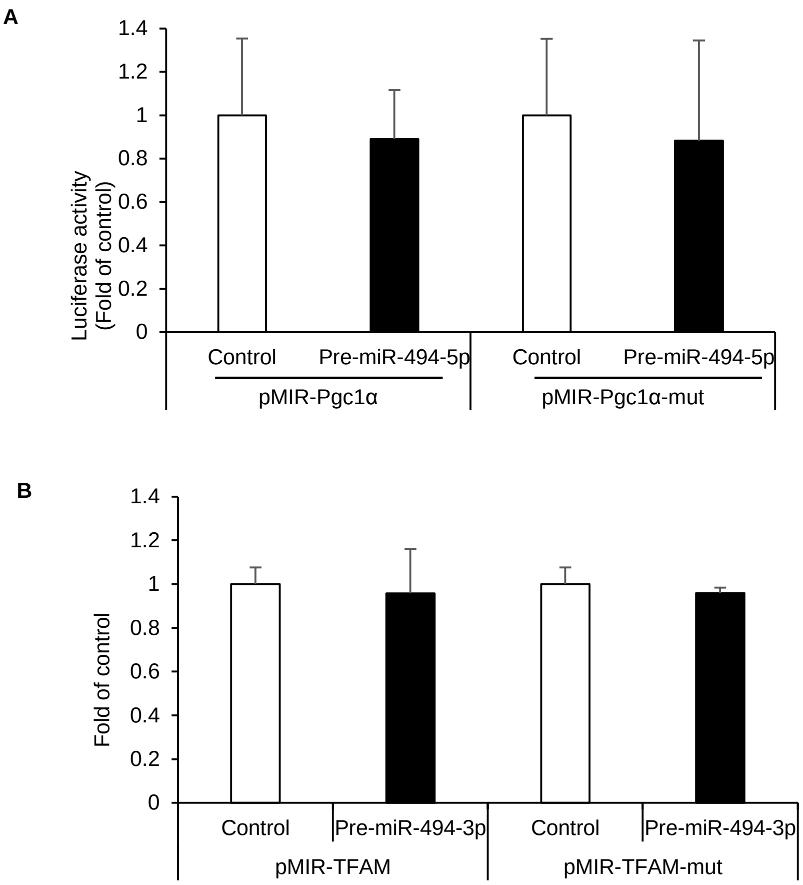

Figure 4. Luciferase reporter assays. **(A)** Luciferase reporters containing the 3`UTR of PGC1α wild-type or mutant 3′-UTR were transfected along with miR-494-5p precursor or scrambled sequence control into beige adipocytes at day 6 for 48 h. n=8. **(B)** Luciferase reporter assays. Luciferase reporters containing the 3`UTR of TFAM wild-type or mutant 3′-UTR were transfected along with miR-494-3p precursor or scrambled sequence control into 3T3-L1 beige adipocytes at day 6 for 48 h. n=4.

Figure 1D

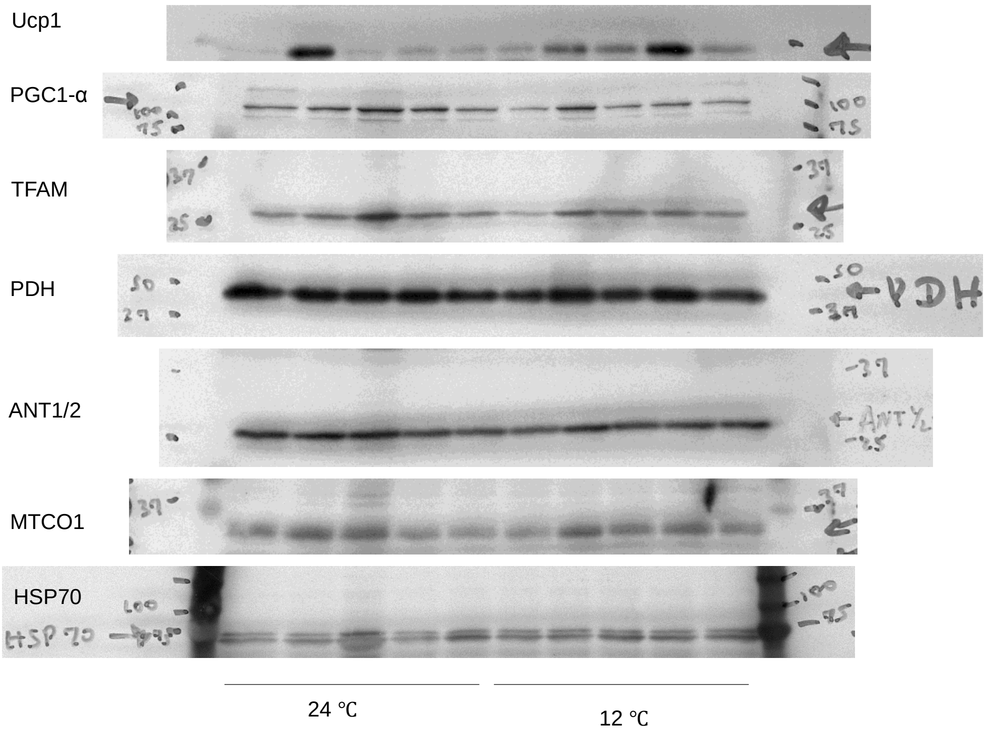

Supplementary Figure 5. Full-length western blot images of figures 1-5.

Figure 1I

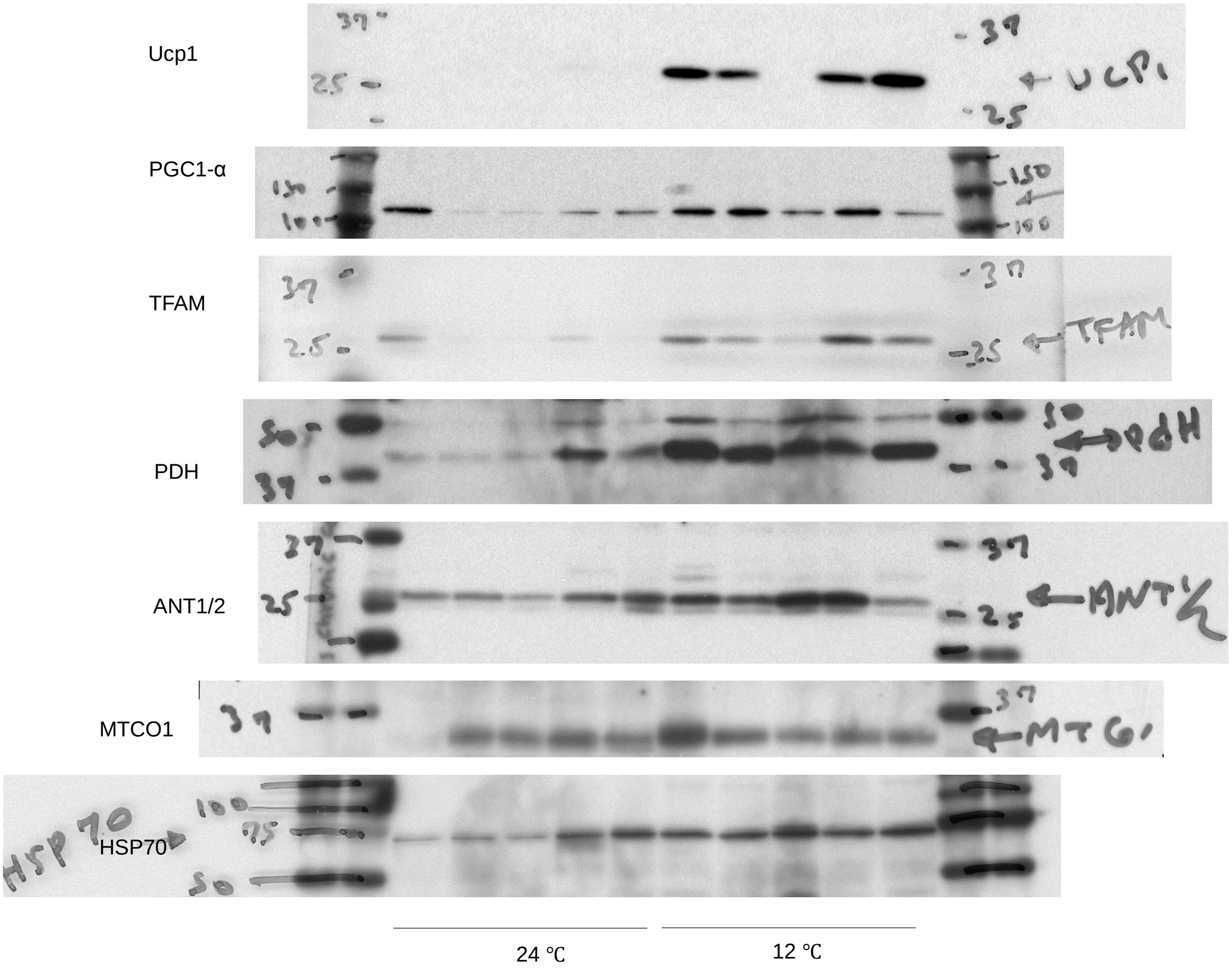

Supplementary Figure 5 continued

Figure 3A

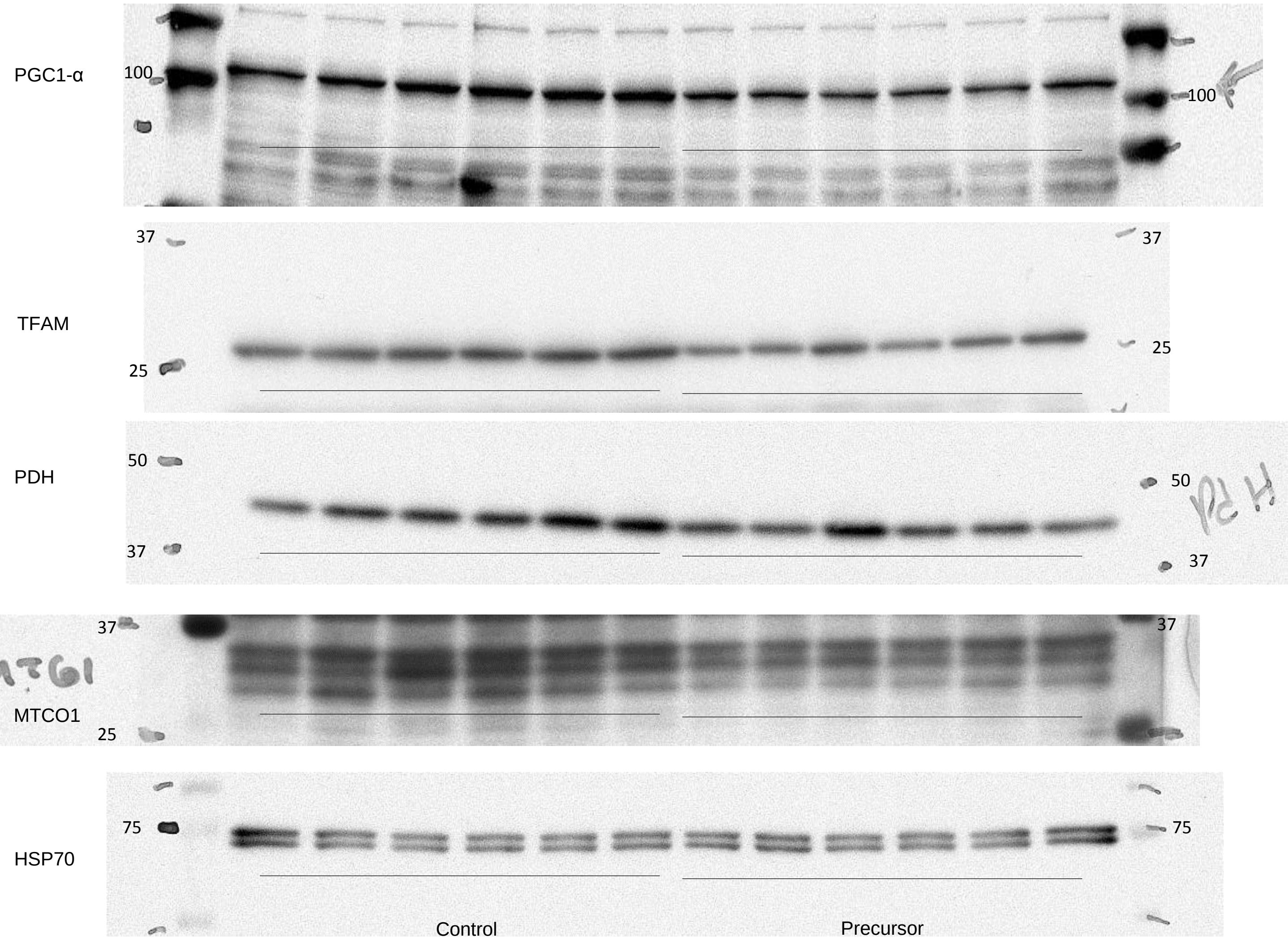

Supplementary Figure 5 continued

Figure 3C

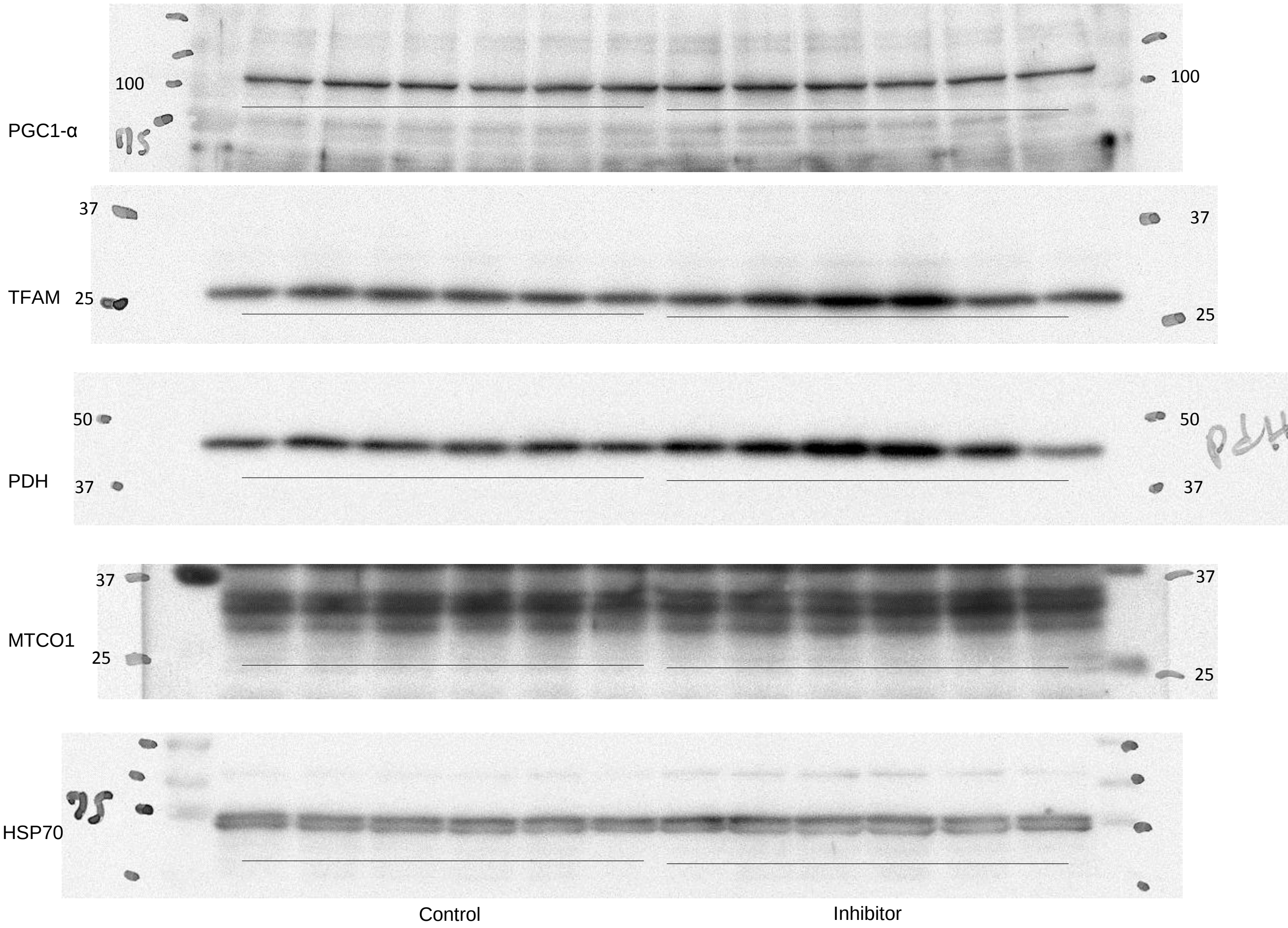

Supplementary Figure 5 continued

Figure 5H

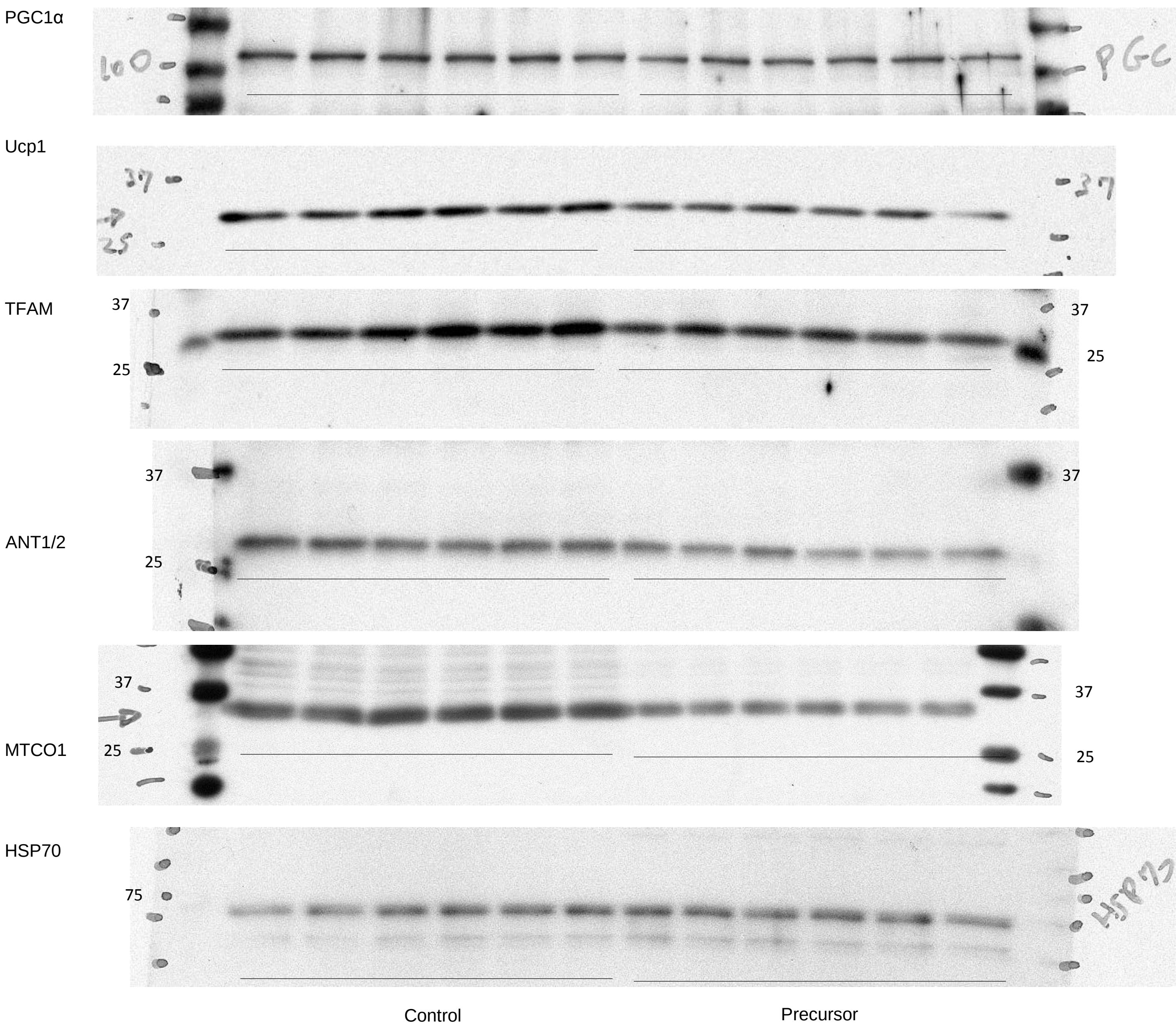

Supplementary Figure 5 continued

Figure 5J

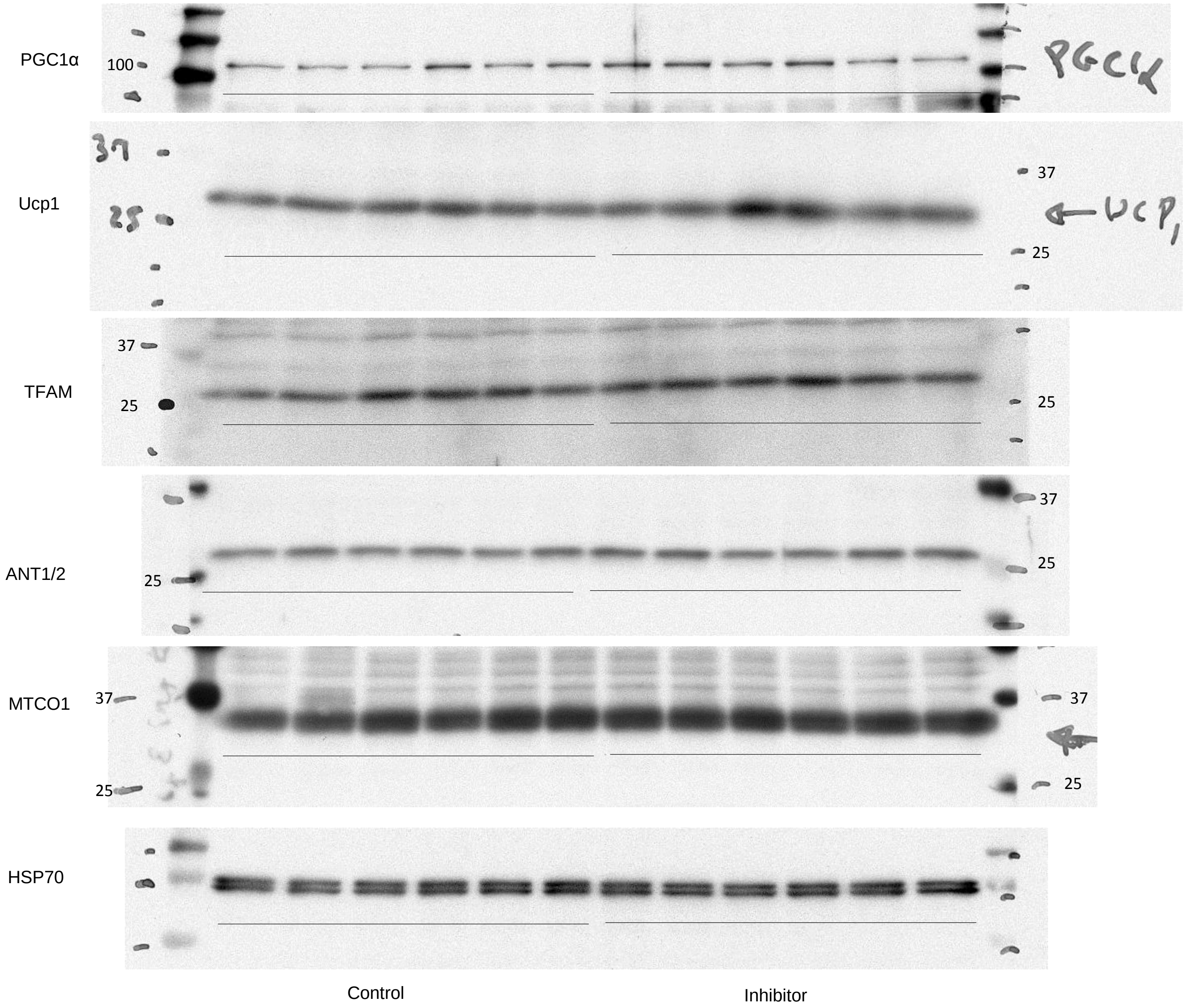

Supplementary Figure 5 continued

Supplementary Figure 3A

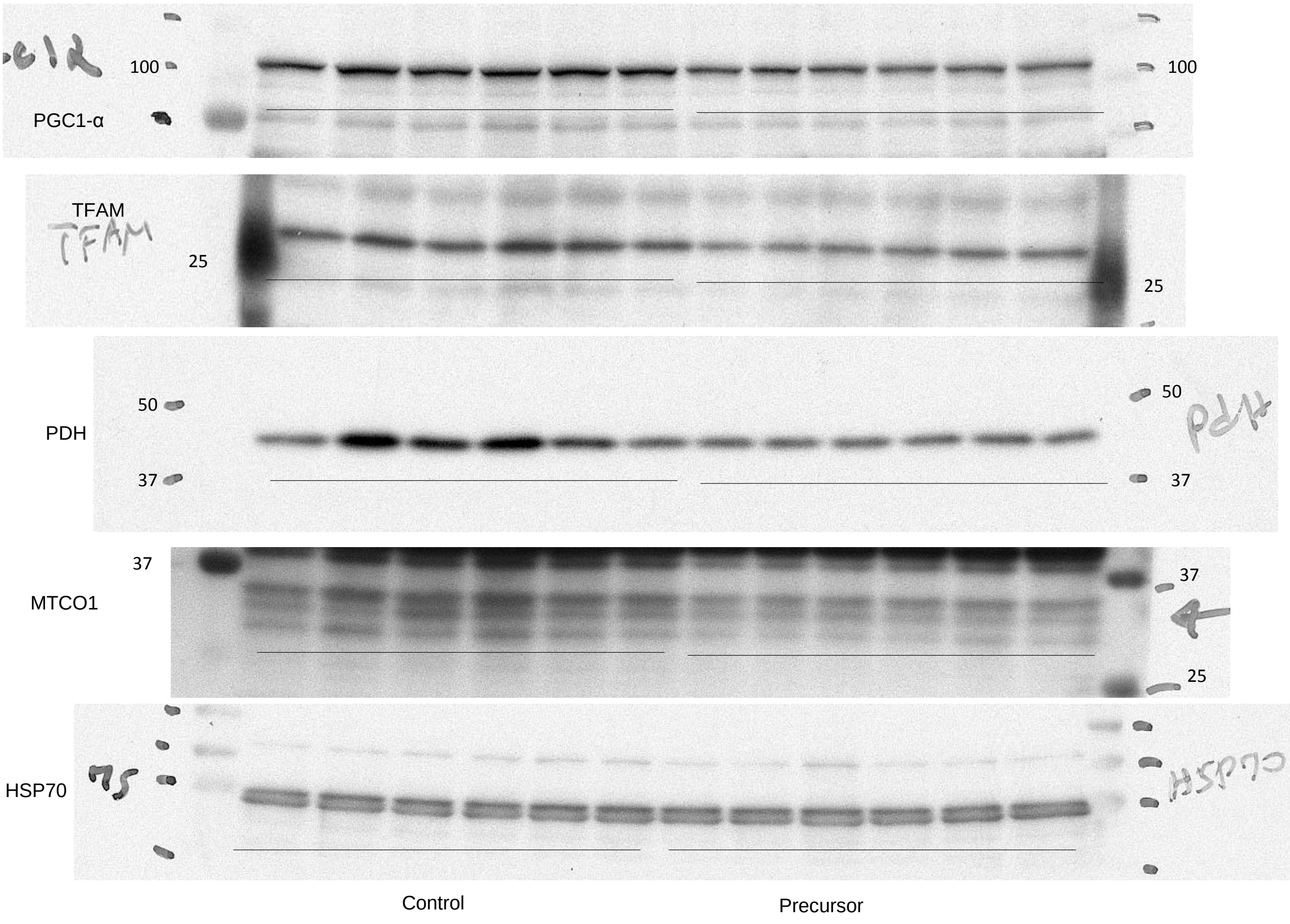

Supplementary Figure 6. Full-length western blot images of supplementary Figure 3.

Supplementary Figure 3C

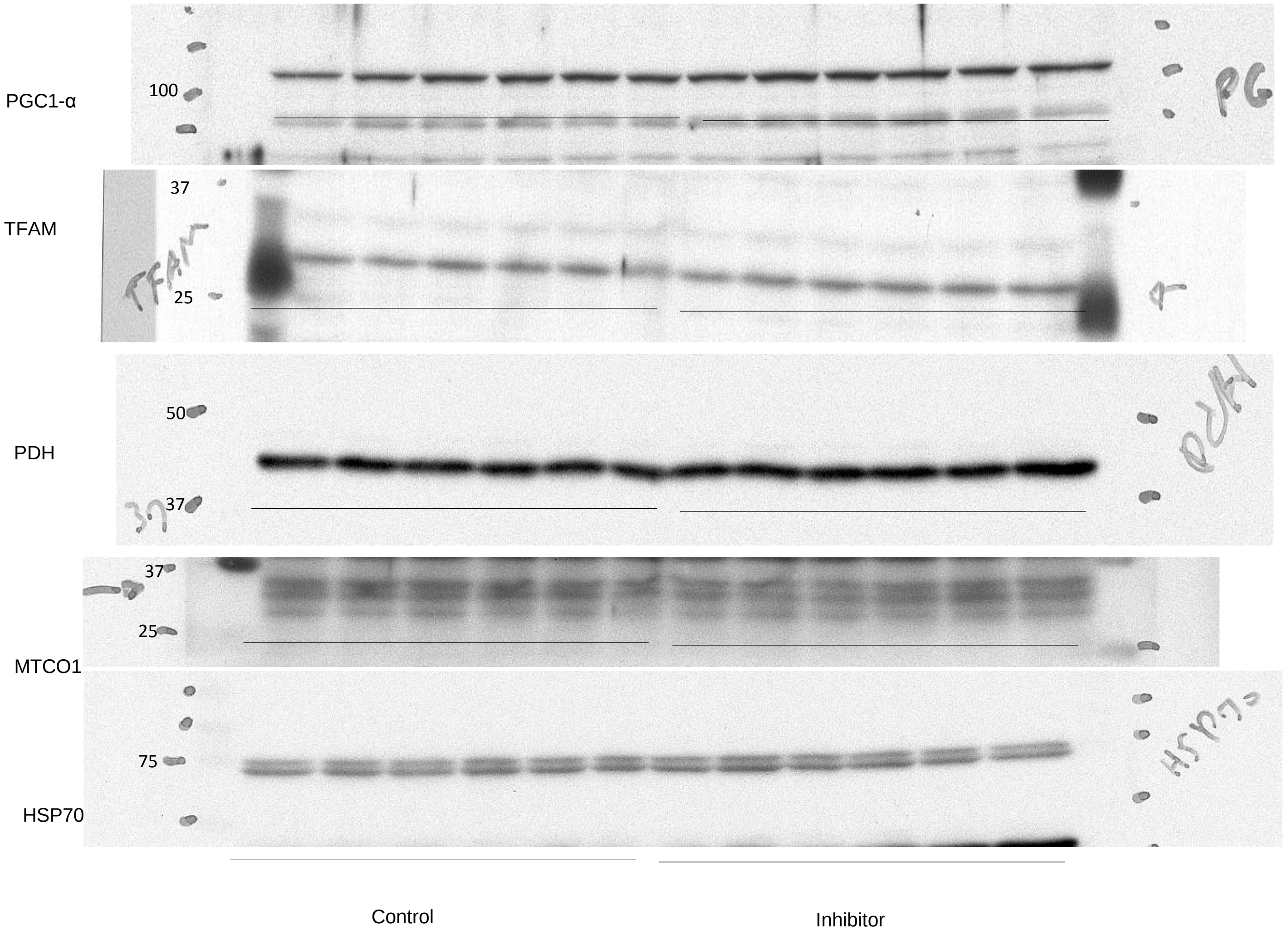

Supplementary Figure 6 continued

.

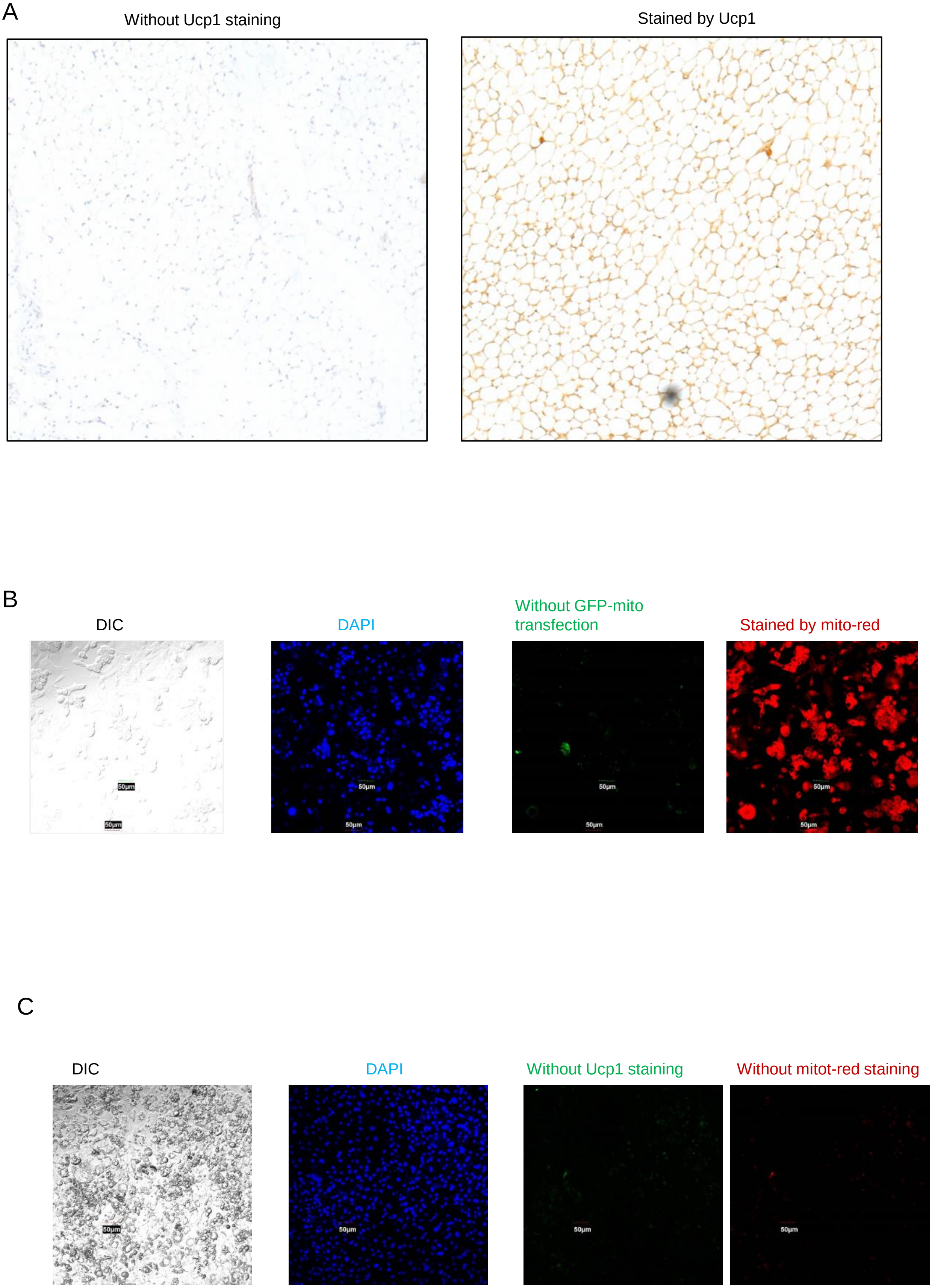

Supplementary Figure 7. Negative controls for Immunohistochemistry and immunofluorescence staining.

(A) Inguinal adipose tissue immunostaining negative control (left without Ucp1 primary antibody and right stained against Ucp1 primary antibody) from 12°C stimulated mouse. **(B)** 3T3-L1 adipocytes immunofluorescence stained with negative control for GFP-mito. **(C)** primary beige adipocytes immunofluorescence stained with negative control for Ucp1 and mitotracker-red.

REFERENCES

1 Asano, H. *et al.* Induction of beige-like adipocytes in 3T3-L1 cells. *J Vet Med Sci* **76**, 57-64 (2014).
